# Reads Binning Improves Alignment-Free Metagenome Comparison

**DOI:** 10.3389/fgene.2019.01156

**Published:** 2019-11-21

**Authors:** Kai Song, Jie Ren, Fengzhu Sun

**Affiliations:** ^1^School of Mathematics and Statistics, Qingdao University, Qingdao, China; ^2^Quantitative and Computational Biology Program, University of Southern California, Los Angeles, CA, United States

**Keywords:** alignment-free methods, metagenomic samples, Markov model, reads binning, beta-diversity

## Abstract

Comparing metagenomic samples is a critical step in understanding the relationships among microbial communities. Recently, next-generation sequencing (NGS) technologies have produced a massive amount of short reads data for microbial communities from different environments. The assembly of these short reads can, however, be time-consuming and challenging. In addition, alignment-based methods for metagenome comparison are limited by incomplete genome and/or pathway databases. In contrast, alignment-free methods for metagenome comparison do not depend on the completeness of genome or pathway databases. Still, the existing alignment-free methods, $d_{2}^{S}$ and $d_{2}^{*}$, which model *k*-tuple patterns using only one Markov chain for each sample, neglect the heterogeneity within metagenomic data wherein potentially thousands of types of microorganisms are sequenced. To address this imperfection in $d_{2}^{S}$ and $d_{2}^{*}$, we organized NGS sequences into different reads bins and constructed several corresponding Markov models. Next, we modified the definition of our previous alignment-free methods, $d_{2}^{S}$ and $d_{2}^{*}$, to make them more compatible with a scheme of analysis which uses the proposed reads bins. We then used two simulated and three real metagenomic datasets to test the effect of the *k*-tuple size and Markov orders of background sequences on the performance of these *de novo* alignment-free methods. For dependable comparison of metagenomic samples, our newly developed alignment-free methods with reads binning outperformed alignment-free methods without reads binning in detecting the relationship among microbial communities, including whether they form groups or change according to some environmental gradients.

## Introduction

Understanding the impact of environmental factors on the composition of microbial communities, along with the effects of microbes on their hosts, is a crucial problem in microbiological studies. Traditional culture-dependent techniques can obtain pure isolates of individual microbes, but such techniques are low-throughput and can capture only a tiny fraction of microbes in a microbial community. With the rapid development of next-generation sequencing (NGS) technology, whole metagenome shotgun sequencing (WMGS) has become a widely used and powerful approach to investigate complex microbial communities (Qin et al., 2010; Qin et al., 2012; Xie et al., 2016; Mehta et al., 2018). Several large scale international metagenomics projects including the Human Microbiome Projects (HMP) (Lloyd-Price et al., 2019) and TARA ocean project (Brum et al., 2015; Sunagawa et al., 2015) have been carried out and most of the metagenomic samples have metadata available. Metagenomic data provide the whole genetic information from microbial communities. A metagenomic sample usually contains millions of short reads, consisting of several hundred of base pairs, and each read is randomly sampled from a genomic region of a microbial genome in the community. Given the massive amount of metagenomic data, computational methods are in great demand to infer the relationships between microbes and environmental factors/hosts. Accurately quantifying the similarities and differences among microbial communities from multiple environments/hosts is one of the most important steps in metagenomic data analysis.

The general approach to analyze metagenomic data is based on alignment methods, such as the Smith-Waterman algorithm (Smith and Waterman, 1981) and BLAST (Altschul et al., 1990), both of which first map NGS reads to known genomes or pathways in existing public protein databases, such as non-redundant (NR), Kyoto Encyclopedia of Genes and Genomes (KEGG), and evolutionary genealogy of genes: Non-supervised Orthologous Groups (eggNOG), and then compare the abundance of different microbial organisms or functional categories between samples (Qin et al., 2010; Muegge et al., 2011; Qin et al., 2012). However, many microbial genomes and gene families are unknown, making it impossible to map all reads to the known genomes or pathways in many environments, in turn making the comparison of metagenomic samples incomplete, as suggested above. Based on the current literature, about 40% of unassigned reads, on average, exist in the human gut microbiome (Qin et al., 2010; Qin et al., 2012), and up to 50% of reads cannot be assigned to reference databases in ocean samples (Marchetti et al., 2012). Apart from alignment-based methods, assembly-based analytical methods reconstruct bacteria genomes by assembling short reads. However, assembly is time-consuming and challenging, especially for metagenomic samples because bacteria genomes can share similar regions, and a short read is not long enough to resolve the ambiguity. These limitations leave alignment-free methods as promising alternative approaches for microbial community comparison by eliminating the requirements of reference sequences or *de novo* assembly.

Although alignment-free methods can be defined as any methods that do not depend on sequence alignment, one of the major types of alignment-free methods is based on the frequencies of *k*-tuples (*k*-words or *k*-mers) as recently reviewed (Song et al., 2014; Zielezinski et al., 2017; Ren et al., 2018). A *k*-tuple is a segment consisting of consecutive nucleotide bases of length *k*. The effectiveness of these alignment-free methods for genome and metagenome comparison was based on the fact that relative *k*-tuple frequencies were similar across different regions of the same genome, but differed between genomes (Karlin et al., 1997). Similarly, the relative *k*-tuple frequencies for closely related genomes would be more similar than those between distantly related genomes. The alignment-free dissimilarity measures, $d_{2}^{S}$ and $d_{2}^{*}$, were developed for high-throughput sequencing data comparison, and they were then used for phylogenetic tree construction (Song et al., 2013), followed by successful applications in the comparison of metagenomic samples (Jiang et al., 2012; Liao et al., 2016) and gene regulatory regions (Song et al., 2013), identification of horizontal gene transfer (Tang et al., 2018b) and virus-host interactions (Ahlgren et al., 2017), and improving contig binning for metagenomes (Wang et al., 2017). Recently, they have also been used to identify the geographic origin of white oak trees (Tang et al., 2018a) and sources of viruses (Li and Sun, 2018). A user-friendly interface for alignment-free genome and metagenome comparison, aCcelerated Alignment-FrEe (CAFÉ) (Lu et al., 2017b), has now been developed. Many other alignment-free methods have been developed including the delta-distance between dinucleotide relative frequencies of different genomes (Kariin and Burge, 1995; Karlin and Mrázek, 1997) and CVTree (Qi et al., 2004a; Qi et al., 2004b). Ren et al. (2018) and Zielezinski et al. (2017) presented the most recent reviews of alignment-free methods for genome and metagenome comparisons and their many applications (Zielezinski et al., 2017; Ren et al., 2018). Zielezinski et al. (2019) recently compared the performance of 74 alignment-free methods for protein sequence classification, gene tree inference, regulatory element detection, genome-based phylogenetic inference, and reconstruction of species trees under horizontal gene transfer, and recombination events. However, the authors did not evaluate their performance on metagenome comparison (Zielezinski et al., 2019).

While the previous alignment-free methods were successful in comparing metagenomic samples, these methods (Jiang et al., 2012; Liao et al., 2016) only considered metagenomics sequencing data as a whole from which to extract *k*-tuple frequencies and calculate their expectations using a common Markov model. However, microbial communities contain thousands of microorganisms and the relative abundance profiles of the microbial communities were shown to change across many environmental factors, such as geographic distance, temperature, oxygen, pH, and biotic factors (Lozupone and Knight, 2007; Steele et al., 2011; Philippot et al., 2013). Different microbial organisms have varied nucleotide frequencies; therefore, it is unreasonable to use only one Markov Chain to model the sequences in a microbial community and to calculate the probability of *k*-tuples. Instead, the present study posits that different Markov models can be used; accordingly, we first organized sequenced bacterial genomes and used them to construct the Markov models. These models were then used for grouping NGS reads into different bins, followed by extracting the *k*-tuples and calculating their expectation in each bin. Markov models have been used extensively for genome modeling (Narlikar et al., 2013), motif discovery (D’haeseleer, 2006), computational gene search (Lomsadze et al., 2005), classification of metagenomic sequences (Brady and Salzberg, 2009) and alignment-free sequence comparison (Chang and Wang, 2011). Next, we extended the definition of our previous alignment-free measures, $d_{2}^{S}$ and $d_{2}^{*}$, to make them more compatible with a scheme of analysis that uses the proposed reads binning datasets. We then used two simulated and three real metagenomic datasets to test the effect of *k*-tuple size and Markov orders of background sequences on the performance of these *de novo* alignment-free methods. For dependable comparison of metagenomic samples, our alignment-free methods with reads binning outperformed alignment-free methods without reads binning in detecting the relationships among metagenomic samples whether they form groups or change according to environmental gradients. For detecting group relationship among samples, the triplet distance between the inferred tree and the gold standard tree is reduced by over 10%. For detecting gradient relationship among the samples, the Pearson correlation coefficient (PCC) between the first principal coordinate and the gradient is increased by 10%. The software is available at https://github.com/songkai1987/MetaBin.

## Materials and Methods

**Figure 1:**
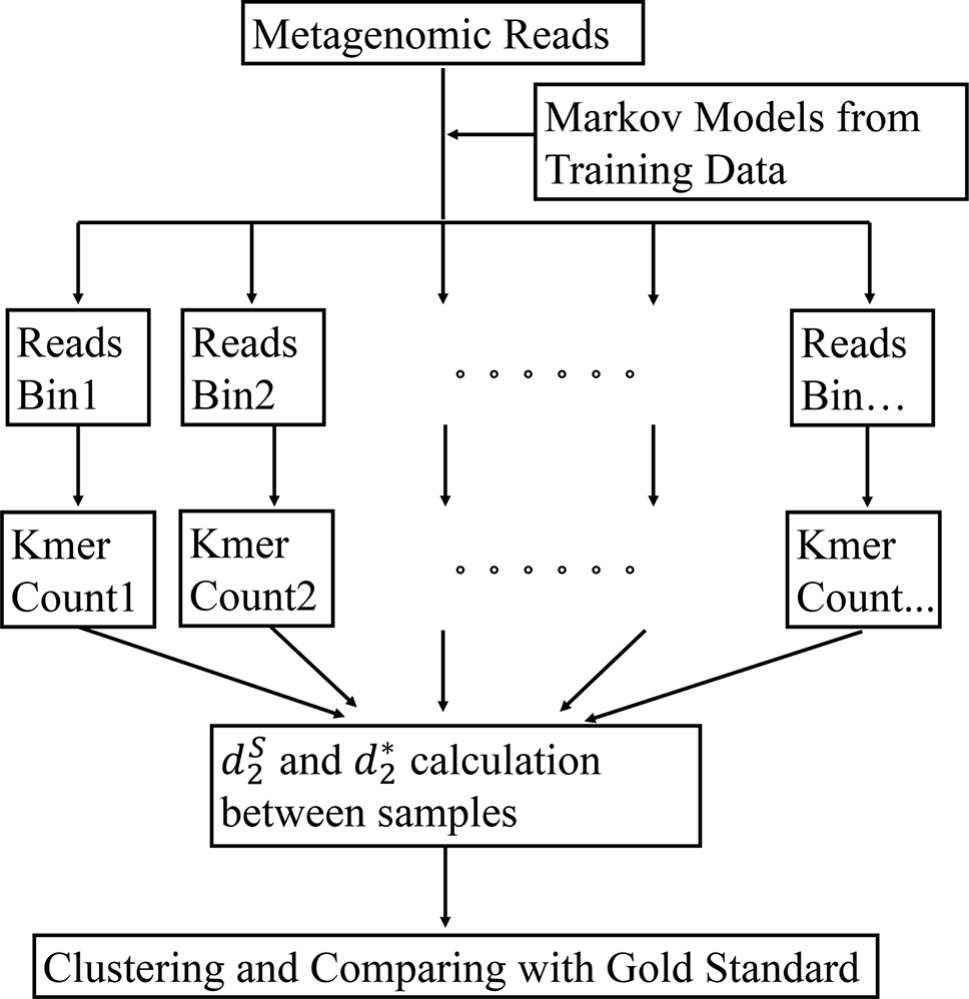
The work flow of our approach. First, the Markov model for each bin is trained using the bacterial genomic sequences. Then, the metagenomic reads are binned to the group under which the sequence has the highest likelihood. The *k*-tuple counts and their expectations are calculated in each bin of the NGS reads. The $d_{2}^{S}$ and $d_{2}^{*}$ are calculated between each pair of samples. Finally, the samples are clustered using the dissimilarity matrix obtained from $d_{2}^{S}$ and $d_{2}^{*}$.

The framework of our method is given in **Figure 1**. First, the bacterial sequences were divided into several bins and a Markov model is used to model the sequences in each bin. Second, each read in the metagenomics samples was assigned to the bin that has the highest probability of generating the sequence. Third, the *k*-tuple counts and their expectations were calculated in each bin of the NGS reads. The $d_{2}^{S}$ and $d_{2}^{*}$ (Eq. 1 and 2) were calculated between each pair of samples. Finally, the samples are clustered using the dissimilarity matrix obtained from $d_{2}^{S}$ and $d_{2}^{*}$ Details of each of the steps are given below.

### The *k*-Tuple Count Vectors and Alignment-Free Comparison Measures

In our previous studies (Jiang et al., 2012; Song et al., 2013), the first step toward comparing metagenomic samples involved counting the number of occurrences of each *k*-tuple. Since a read could be from the forward or reverse strand of a genome, we considered each read together with its complement when calculating the occurrences of each *k*-tuple. Thus, for metagenomic data, we have a finite alphabet set *S={A,C,G,T}* and consider all possible *k*-tuples in the reads of metagenomic samples. Let $X=(X_{1},X_{2},\text{...},X_{4_{}^{k}})$ and $Y=(Y_{1},Y_{2},\text{...},Y_{4_{}^{k}})$ be the *k*-tuple count vectors of two metagenomic samples *X* and *Y*, respectively. Then, we define the centralized count variables by using Markov model-based expectation as

$${\bar{X}}_{i}=X_{i}-n_{X}p_{X,i}$$

$${\bar{Y}}_{i}=Y_{i}-n_{Y}p_{Y,i}$$

where *n* *_X_* is the total count of *k*-tuples, and *p* *_X,i_* is the probability of *i*-th *k*-tuple under the Markov model of order *r*. The idea behind subtracting the expected *k*-tuple count from the observed count is that the *k*-tuples responsible for the similarity between microbial communities will stand out after subtraction. Then, the two measures $d_{2}^{S}$ and $d_{2}^{*}$ can be defined as

$$D_{2}^{S}(X,Y)=\sum_{i=1}^{4^{k}}\frac{{\bar{X}}_{i}{\bar{Y}}_{i}}{\sqrt{{\bar{X}}_{i}^{2}+{\bar{Y}}_{i}^{2}}}$$

(1) $$d_{2}^{S}(X,Y)=\frac{1}{2}\left(1-\frac{D_{2}^{S}(X,Y)}{\sqrt{\sum_{i=1}^{4^{k}}\frac{{\bar{X}}_{i}^{2}}{\sqrt{{\bar{X}}_{i}^{2}+{\bar{Y}}_{i}^{2}}}\sum_{i=1}^{4^{k}}\frac{{\bar{Y}}_{i}^{2}}{\sqrt{{\bar{X}}_{i}^{2}+{\bar{Y}}_{i}^{2}}}}}\right)$$

and

$$D_{2}^{*}(X,Y)=\sum_{i=1}^{4^{k}}\frac{{\bar{X}}_{i}{\bar{Y}}_{i}}{\sqrt{n_{X}p_{X,i}}\sqrt{n_{Y}p_{Y,i}}}$$

(2) $$d_{2}^{*}(X,Y)=\frac{1}{2}\left(1-\frac{D_{2}^{*}(X,Y)}{\sqrt{\sum_{i=1}^{4^{k}}\frac{{\bar{X}}_{i}^{2}}{n_{X}p_{X,i}}\sum_{i=1}^{4^{k}}\frac{{\bar{Y}}_{i}^{2}}{n_{Y}p_{Y,i}}}}\right)$$

The first statistic $D_{2}^{S}$ is based on the observation by Shepp (Shepp, 2006) that for two independent normal random variables *X* and *Y* with mean zero, $XY/\sqrt{X^{2}+Y^{2}}$ is also normally distributed. The second statistic $D_{2}^{*}$ is motivated by Pearson correlation where the mean and variance of each tuple are calculated based on Poisson distribution assumption for the *k*-tuples. When the two samples are more similar, the *k*-tuple frequency profiles are more similar and the values of $D_{2}^{S}$ and $D_{2}^{*}$ are higher. The ranges of $D_{2}^{S}$ and $D_{2}^{*}$ can depend on the nucleotide frequencies. In order to make their range independent of nucleotide frequencies, we normalize them to dissimilarities, $d_{2}^{S}$ and $d_{2}^{*}$, respectively, so that they have a range between 0 and 1 according to the Cauchy inequality. When two samples are similar, the values of $d_{2}^{S}$ and $d_{2}^{*}$ are close to 0.

### The Alignment-Free Measures Based on a Mixture of Markov Models Learned From Reads Bins

Metagenomic samples consist of a mixture of many different microbial genomes; thus, it is unreasonable to expect that all these reads can be modeled using only one single Markov model for each sample. To address this difficulty, we first group these reads into different bins. Then, we count the *k*-tuple vectors and obtain the expectation of each *k*-tuple for the reads in each bin individually.

We used the bacterial genomic sequences to train the Markov models. First, we calculated the guanine-cytosine (GC) frequency of each bacterial genomic sequence and then grouped these bacterial genomic sequences into different bins using the quantiles of the GC frequency distribution. Each bin has the same number of bacterial genomes. The Markov model for each bin was then constructed using the *k*-tuple vectors counted from all the genomic sequences in that bin. For a set of genomic sequences in a bin, let *X* *_w_* be the count of *k*-tuple *w* of all these genomes and their complementary sequence. The Markov model of order *r* is defined as a 4*^r^*×4 matrix of transition probabilities. The transition probabilities can be estimated based on the *r*-tuples and (*r*−1)-tuples, and the estimated probability of observing nucleotide *w* *_r_*_+1_ given preceding nucleotides *w*_1_ *w*_2_···*w* *_r_* is $P_{M}(w_{r+1}|w_{1}w_{2}\cdots w_{r})=\frac{X_{w_{1}w_{2}\cdots w_{r}w_{r+1}}}{X_{w_{1}w_{2}\cdots w_{r}}}$, where $X_{w_{1}w_{2}\cdots w_{r}}$ and $X_{w_{1}w_{2}\cdots w_{r}w_{r+1}}$ are the counts of *r*-tuple *w*_1_ *w*_2_···*w* *_r_* and (*r*+1)-tuple *w*_1_ *w*_2_···*w* *_r_* *w* *_r_*_+1_, respectively.

Once we have *C* different Markov models of order *r*, $\left(M_{r}^{1},M_{r}^{2},\cdots ,M_{r}^{\text{C}}\right)$, to model the bacterial genomic sequences, we classify the reads in a metagenomic sample to the bins with the highest log-likelihood scores. In particular, suppose *Y*=*y*_1_ *y*_2_···*y* *_N_* represents a read of length N in a metagenomic sample; then, the log-likelihood of the read under the Markov chain *M* *_r_* could be calculated as

$$\mathrm{LL}(Y|M_{r})=\sum_{i=1}^{N-r}\log P_{M_{r}}(y_{i+r}|y_{i}y_{i+1}\cdots y_{i+r-1})$$

Then, the classification of read could be defined as the model having the largest probability, or

(3) $$l=\underset{c=1,L,C}{\arg\max}\mathrm{LL}(Y|M_{r}^{c})$$

where *λ* is the predicted bin to which the read belongs.

Next, we calculate the *k*-tuple count and its expectation in each bin of NGS reads. The centralized count variables by using Markov model-based expectation such that all *C* bins are combined are as follows:and

(4) $${\bar{X}}_{w}=\sum_{c=1}^{C}(X_{w}^{c}-n_{X}^{c}p_{X,w}^{c})$$

$${\bar{Y}}_{w}=\sum_{c=1}^{\text{C}}(Y_{w}^{c}-n_{Y}^{c}p_{Y,w}^{c})$$

where *c* represents the calculation based on the *c*-th bin. Therefore, the two measures $d_{2}^{S}$ and $d_{2}^{*}$, could be defined using the new version of ${\bar{X}}_{w}$ and ${\bar{Y}}_{w}$.

### Comparison With Other Reads Binning Approaches Without Reference Genomes

In addition to the above reads binning method, we also considered creating reference-free reads binning by first assembling reads into contigs and grouping contigs into bins. Metagenomic reads are then classified to different bins based on their similarity to the contigs in those bins. MetaSPAdes (Bankevich et al., 2012; Nurk et al., 2017) was used to cross-assemble the reads in the simulated datasets using the default setting. Contig coverages [Fragments Per Kilobase per Million reads (FPKMs)] were determined by mapping reads with Bowtie2 (Langmead and Salzberg, 2012), using the default settings, and were averaged for each bin. Sequence COmposition, read CoverAge, CO-alignment, and paired-end read LinkAge (COCACOLA) (Lu et al., 2017a) and MetaBAT (Kang et al., 2015) were used to cluster these assembled contigs (≥500 bp) based on sequence tetra-nucleotide frequencies and contig coverages normalized by contig length and number of mapped reads in samples, respectively. MetaBAT performed better than other approaches in the CAMI study (Meyer et al., 2018). The simulated reads were mapped to the set of contigs using Burrows-Wheeler-Aligner (BWA) software (Li and Durbin, 2009) to obtain the classification labels. The unmapped reads were binned together as an extra bin. We calculated the *k*-tuple counts and their expectation in each bin and then calculated the values of $d_{2}^{S}$ and $d_{2}^{*}$.

### Comparison With Other Reads Binning Approaches With Reference Genomes

We compared our method with two reference genome-based reads binning approaches, Kraken (Wood and Salzberg, 2014) and MBMC (Wang et al., 2016), to classify the metagenomic reads. Kraken is a program for assigning taxonomic labels to metagenomic DNA sequences and it has been shown to perform better than other binning approaches, such as Megablast (Chen et al., 2015), PhymmBL (Brady and Salzberg, 2009), NBC (Rosen et al., 2008) and MetaPhlAn (Segata et al., 2012). The core of Kraken is a database consisting of *k*-tuples and the lowest common ancestor (LCA) of all organisms whose genomes contain the *k*-tuples. Sequences are classified by querying the database for each *k*-tuple in a sequence, and then using the resulting set of LCA taxa to determine an appropriate label for the sequence. To compare with our method, the 100 bacterial genomes in simulations were used to construct the genome library for *k*-tuples and their LCAs in Kraken. MBMC is a recent approach for binning reads by measuring the similarity of reads to the trained Markov chains for different taxa using the ordinary least squares (OLS) method. Similarly, the 100 bacterial genomes in simulations were also used for constructing the Markov chains, respectively. Each of the two approaches was then used to classify reads into different bins individually. We calculated the *k*-tuple counts and their expectations in each bin to then calculate the values of $d_{2}^{S}$ and $d_{2}^{*}$.

### Beta-Diversity Analysis and Evaluation Methods

Detection of group relationships among metagenomic samples and the identification of external gradients driving shifts in microbial community structure are two major types of analytical tasks in microbial community comparison. Therefore, we evaluated the performance of our new alignment-free measures in metagenomic sample comparison by assessing how well they would detect the known group relationships or identify known environmental gradients.

For clustering analysis, we used the unweighted pair-group method with arithmetic means (UPGMA) algorithm (Murtagh, 1984) to cluster metagenomic samples based on the pairwise dissimilarity defined using our alignment-free measures, and then we compared the clustering tree with the true group relationship among the samples. We used the R package “phangorn” (Schliep, 2011) for clustering samples given the input of the pairwise dissimilarity matrix. The triplet distance was used to measure the distance between the tree built using our methods and the ground truth. Triplet distance was proposed by (Critchlow et al., 1996) as a measure for the distance between two rooted bifurcating phylogenetic trees, and it can be used for measuring the distance between binary (Critchlow et al., 1996) or non-binary trees (Bansal et al., 2011). This measure first decomposes the topologies of the input trees into triplets, i.e., all three-element subsets of the set of leaves, and then computes how many triplets of the two trees have different topologies. Because triplets are the basic building blocks of rooted and unrooted trees, in the sense that they are the smallest topological units that completely identify a phylogenetic tree, triplet-based distances provide a robust and fine-grained measure of the dissimilarities between trees (Bansal et al., 2011). This was finally developed into the TreeCmp toolbox (Bogdanowicz et al., 2012).

For the study of gradient relationships among the samples, the shift of metagenomic samples is visualized by PCoA (Principal Coordinates Analysis), which is a multidimensional scaling (MDS) method that converts between-sample dissimilarity matrix into two-dimensional, or three-dimensional, ordinates of samples and arranges the samples in ordinate space. We used the MASS package in R for PCoA (Anderson, 2003). Then, the influence of environmental gradient(s) on microbial communities could be investigated by calculating correlation, such as PCC, between the first principal coordinate and the gradient axis. In this way, the performance of the alignment-free methods could be evaluated, as long as the gradient driving microbial communities is known.

### Simulated Metagenomic Datasets

We simulated two NGS metagenomic datasets using Next-generation Sequencing Simulator for Metagenomics (NeSSM) (Jia et al., 2013), which supports single-end and paired-end sequencing for both 454 and Illumina platforms, with paired-end short reads of length 150 bp in an Illumina MiSeq setting mode based on abundance profiles. Since 1) the database for reference genome is not complete and 2) new genomes can be discovered in the future, we mimic the situation by splitting the reference genomes by May 2015 such that the genomes before this date were used for training the Markov chain models, and the genomes after this date were used to simulate the metagenomic datasets for testing. A set of 100 bacterial species randomly sampled from the 5,865 sequenced bacterial reference genomes from NCBI was used for simulation (**Table S1**). We designed two sets of metagenomic samples representing the two types of relationships among samples as has been done in (Jiang et al., 2012): the group relationship involving species abundance levels of the samples belonging to different groups and the gradient relationship involving species abundance levels that change continuously with some environmental variables, such as temperature or location.

In Simulation 1, we simulated 60 samples belonging to three groups. For each group, we randomly chose 100 genomes and assigned the i-th genome with relative abundance generated from the power-law (Zipf’s) distribution as $f(m;\alpha ,N)=\frac{1/m^{\alpha }}{\sum_{n=1}^{N}1/n^{\alpha }}$, m = 1, 2, …, N, where N = 100, and α is the value of the exponent characterizing the distribution. We set α=0.3and generated three relative abundance vectors from power-law distribution by randomly ordering the 100 genomes as the centers of the three groups. We next added to each component the absolute value of a Gaussian noise with mean zero and variance equal to 10 times each component and then renormalized each component to sum to 1. Each relative abundance vector was randomized and renormalized 20 times, and a total of 60 relative abundance vectors were obtained. Then, we used the relative abundance vectors to simulate 60 metagenomic samples.

In **Simulation 2**, we generated 20 samples consisting of the same 100 genomes, and the relative abundance vector of 100 genomes was generated by the power law (Zipf’s law) distribution as defined in the above simulation. In order to mimic the gradient model, the relative abundance vector shifts along a gradient axis of *α*from 0.30 to 0.70 by step 0.02. Again, absolute values of Gaussian noises were added to each component of the 20 abundance vectors with mean 0 and standard deviation equal to the value of that component. The vectors were renormalized after adding the noises. We generated 20 metagenomic samples according to these relative abundance vectors using NeSSM.

In all simulations, we generated datasets at two sequencing depths: 0.1M and 0.5M sequencing reads per sample. At each setting, we generated 30 duplicated datasets to simulate possible stochastic effects in real NGS data.

### Real Metagenomic Datasets

We analyzed three real shotgun metagenomic sequencing datasets published in recent years. For real datasets, we used all genomic sequences to train the Markov models.

### The Human Gut Datasets

The first dataset includes 107 fecal microbiome samples from Asia (Kurokawa et al., 2007; Qin et al., 2012), Europe (Qin et al., 2010) and North America (Turnbaugh et al., 2009). The dataset includes samples from two countries (China and Japan, n = 45 and 13) in Asia, two countries (Denmark and Spain, n = 21 and 10) in Europe, and one country (USA, n = 18) in North America. The accession numbers for the samples are given in **Table S2** in the supplementary material. We investigated this dataset at two levels. First, we considered the samples from different continents and studied the relationships among these samples. Then, we considered the samples from different countries and studied the relationships among these samples with respect to their countries of origin.

### The Human Microbiome Datasets

The second dataset includes 60 microbiome samples from four body sites: buccal mucosa, supragingival plaque, tongue dorsum and stool (Lloyd-Price et al., 2017). The accession numbers for the samples are given in **Table S3** in the supplementary material. We investigated the relationships among these microbial samples from different body sites.

### The Soil Metagenomic Dataset

This dataset includes 16 soil metagenomic samples from 16 sites: 3 from hot deserts, 6 from Antarctic cold deserts, and 7 from temperate and tropical forests, a prairie grassland, a tundra, and a boreal forest (Fierer et al., 2012). The accession numbers of these samples are given in **Table S4** in the supplementary material. The sites span a wide range of ecologically distinct microbiomes to examine how cold desert soils compare with those from hot deserts, forests, prairie, and tundra. We investigated the relationships among these different ecologically distinct microbiomes and explored their relationship to environmental factors, such as pH values.

## Results

We conducted a series of computational experiments including both intensive simulations and real dataset analyses to study the effect of *k*-tuple-based alignment-free methods with or without reads binning on identifying group and gradient relationships of metagenomic samples. To accomplish this, we first simulated two types of metagenomic datasets to investigate the performance of our newly developed alignment-free measures $d_{2}^{S}$ and $d_{2}^{*}$, and the effect of several factors, such as the *k*-tuple size and Markov orders of background sequences, on their performance. The simulated datasets were generated based on sampling reads from one hundred bacterial genomes randomly chosen from those detected after June 2015 with different abundance levels. The genomes discovered before May 2015 were used for training the Markov models for reads binning. We binned bacterial genomes by their GC content, and then, for each bin, we trained a Markov chain to model sequences in that bin. For reads in the simulated metagenomic samples, we classified them into different bins based on their likelihood evaluated under the corresponding Markov models [Eq. (3)]. The *k*-tuple frequency vectors were counted and normalized individually for each group [Eq. (4)]. Finally, the pairwise alignment-free dissimilarities, $d_{2}^{S}$ and $d_{2}^{*}$, were computed between samples based on Eq. (1, 2), and β-diversity analysis was implemented to evaluate how well the true underlying relationship among samples could be recovered by our method. We also compared our newly developed methods with the original version of the alignment-free measures in (Jiang et al., 2012; Song et al., 2013) which were based on *k*-tuples, but without reads binning. In addition, we also compared our approach with two reference-free binning methods, COCACOLA and MetaBAT, and two other reference-based binning methods, Kraken and MBMC.

### Simulation 1: Detecting Group Relationships Among Metagenomic Samples

In some situations, metagenomic samples may form different groups. For example, gut samples may group based on diet, and soil samples may group based on locations. In order to evaluate the ability of dissimilarity measures to detect such group relationships, we simulated datasets of 60 metagenomic samples belonging to three different groups (20 samples in each group) similar to the simulation design of (Jiang et al., 2012). Each sample was generated by simulating NGS reads from a mixture of 100 bacterial genomes detected after June 2015 with different abundance levels (see Materials and Methods for details).

**Figure 2:**
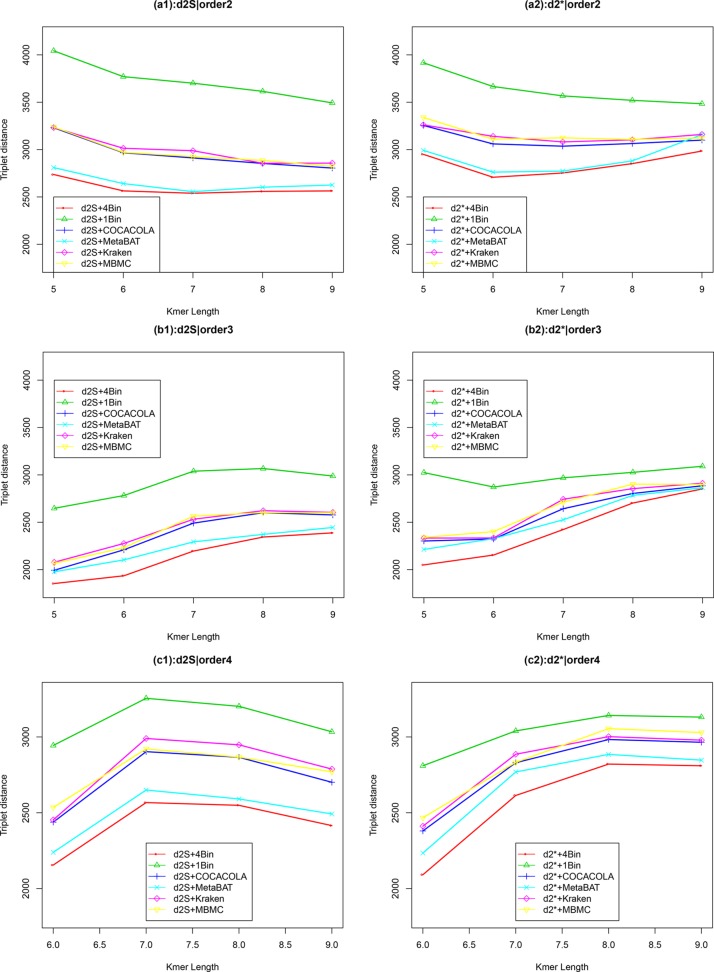
The relative performance (triplet distance) of various reads binning methods in recovering group relationships of the metagenomic samples for Simulation 1 at sequencing depth of 500,000 NGS paired-end reads. The background sequence Markov orders were two (a1, a2), three (b1, b2), and four (c1, c2). The dissimilarity measures $d_{2}^{S}$ and $d_{2}^{*}$ with binning into 4 bins outperform other binning methods in most situations. The corresponding figures based on Markov order zero and one are presented as **Figure S2** in **Supplementary Material**.

**Figure 3:**
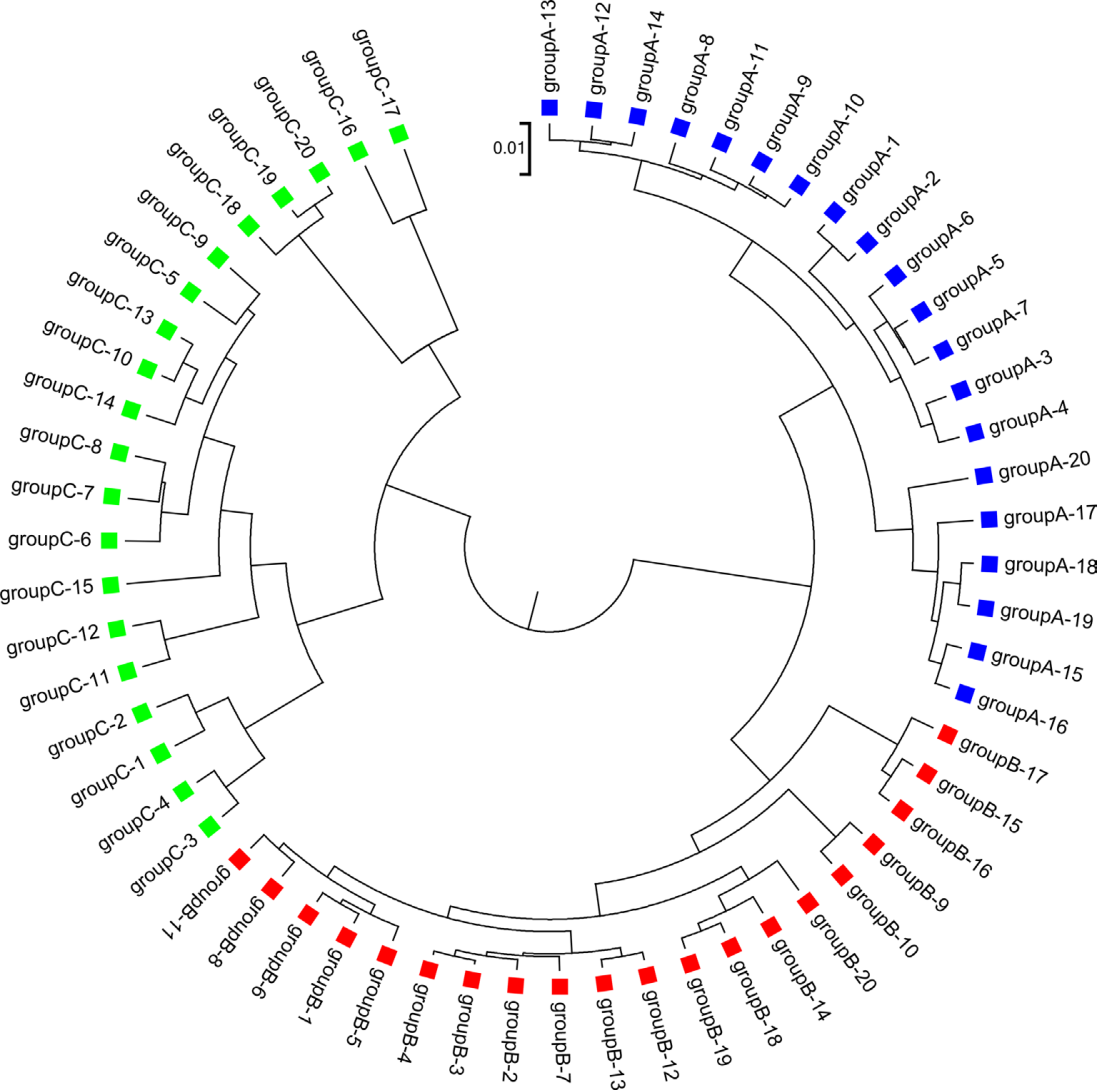
The best clustering tree for the 60 simulated metagenomic samples in Simulation 1 based on the newly developed dissimilarity measure $d_{2}^{S}$ with reads grouped to 4 bins, tuple size *k* = 5, and background sequence Markov order = 3.

We applied our newly developed alignment-free measures $d_{2}^{S}$ and $d_{2}^{*}$ to detect group relationships of the 60 samples by clustering analysis. We studied various factors, including the number of bins, the order of the Markov model for the background sequences, the tuple size *k*, and sequencing depth, all affecting the performance of $d_{2}^{S}$ and $d_{2}^{*}$ in recovering the group relationships among the samples. **Figure 2** showed that both $d_{2}^{S}$ and $d_{2}^{*}$ dissimilarity measures with reads binning outperform the original versions without reads binning. The best clustering result with the smallest triplet distance is obtained by $d_{2}^{S}$ with reads binning using tuple size *k* = 5, Markov order 3 (**Figure 3**). To test if the lowest triplet distance is statistically significantly lower than the second lowest triplet distance, we generated 10 duplicated datasets to simulate possible stochastic effects in real NGS data and obtained the triplet distances between the inferred clustering and the reference cluster for each duplication. Using paired t-test, the resulting one side p-value is less than 0.0005 indicating that the lowest and the second lowest triplet distances are statistically significantly different. In **Table 1**, we fixed the tuple size at 5 for $d_{2}^{S}$ and $d_{2}^{*}$, and compared the effect of reads binning number on recovering group relationships. The results showed that alignment-free methods without reads binning had the largest values of triplet distance, i.e., the worst performance, compared to alignment-free methods with reads binning from 2 to 5 bins, which improved performance. Reads binning from 3, 4, or 5 bins could achieve similar performance. The simulations using a relatively shallow sequencing with 100,000 paired-end reads also gave results similar to those of deeper sequencing with 500,000 paired-end reads (**Figure S3**).

**Table 1:** The triplet distances between the reference and the clustering trees using various numbers of bins for the reads with tuple size *k* = 5 and background sequence Markov order from 0 to 3 for Simulation 1 at sequencing depth of 500,000 next-generation sequencing paired-end reads.

		No binning	2 bins	3 bins	4 bins	5 bins
$d_{2}^{S}$	order 0	3,535	2,634	2,635	2,634	2,633
	order 1	4,123	3,472	3,593	3,619	3,666
	order 2	4,043	2,867	2,846	2,737	2,726
	order 3	2,647	**1,852**	1,856	**1,853**	1,875
$d_{2}^{*}$	order 0	3,723	2,629	2,668	2,676	2,663
	order 1	4,183	3,833	3,977	3,992	4,042
	order 2	3,893	2,987	2,971	2,950	2,943
	order 3	2,986	2,087	2,020	2,050	2,045

The two lowest triplet scores are in boldface.

We next investigated the effects of sequencing errors on the performance of our methods and the results are shown in **Figure S1(a, b)** in the supplementary material. As expected, the sequencing errors could affect the accuracy of the reads assembly and contig binning, which in turn affect the clustering results. The triplet distance did not increase with sequencing error rate significantly until the sequencing error rate equals to 0.05 (**Figure S1**, p-value < 0.05 for t-tests). For reference, the sequencing error rates of Illumina and 454 platforms are ∼0.001 or 0.01, respectively (Glenn, 2011), so sequencing errors only slightly impact the performance of the measures at the reported error rates for the NGS technologies.

We next considered other reference-independent and reference-dependent ways to construct Markov chain models. We cross-assembled the reads from the 60 metagenomic samples and used COCACOLA (Lu et al., 2017a) and MetaBAT (Kang et al., 2015), two reference-independent contig binning methods, to bin these contigs, respectively. We also used two reference-based reads binning methods, Kraken (Wood and Salzberg, 2014) and MBMC (Wang et al., 2016), based on bacterial genomes to group the metagenomic reads into different bins. Then, Markov chain models were constructed for each contig bin, and reads were then classified in the same way to each contig bin based on their likelihood under different Markov models. We compared these reads binning schemes with our approach. **Figure 2** show the corresponding results. It can be seen that all these reads binning schemes are better than the original version without any reads binning procedure, but they do not perform as well as the above scheme based on binning from Markov chains.

### Simulation 2: Revealing Environmental Gradients From Metagenomic Samples

The second simulation experiment was designed to evaluate the effectiveness of the alignment-free methods for analyzing gradient variation of microbial communities. A set of 20 metagenomic samples was generated by simulating NGS reads from 100 bacterial species also used in the above simulations with varying abundance levels. We designed the proportion of the 100 genomes to vary from sample 1 to sample 20 in a way that would mimic gradient variation across the samples, and then, we evaluated the performance of the alignment-free methods in terms of revealing such gradient variations from the metagenomics data.

Dissimilarity matrices were calculated using the alignment-free methods with different *k*-tuple sizes and Markov orders of background sequences as above. PCoA (Anderson, 2003), an effective approach to display β-diversity among multiple samples, mapped the 20 samples to a two-dimensional space. Then, the PCC was calculated between the first principal coordinate (PC1) given by PCoA and the predetermined gradient axis built into the simulation model. PCC can be taken as an index of how well the alignment-free method reveals the gradient variation in samples (see *Materials and Methods* for details). A higher PCC indicates better performance of the dissimilarity measure in recovering the gradient among the microbial samples.

**Figure 4:**
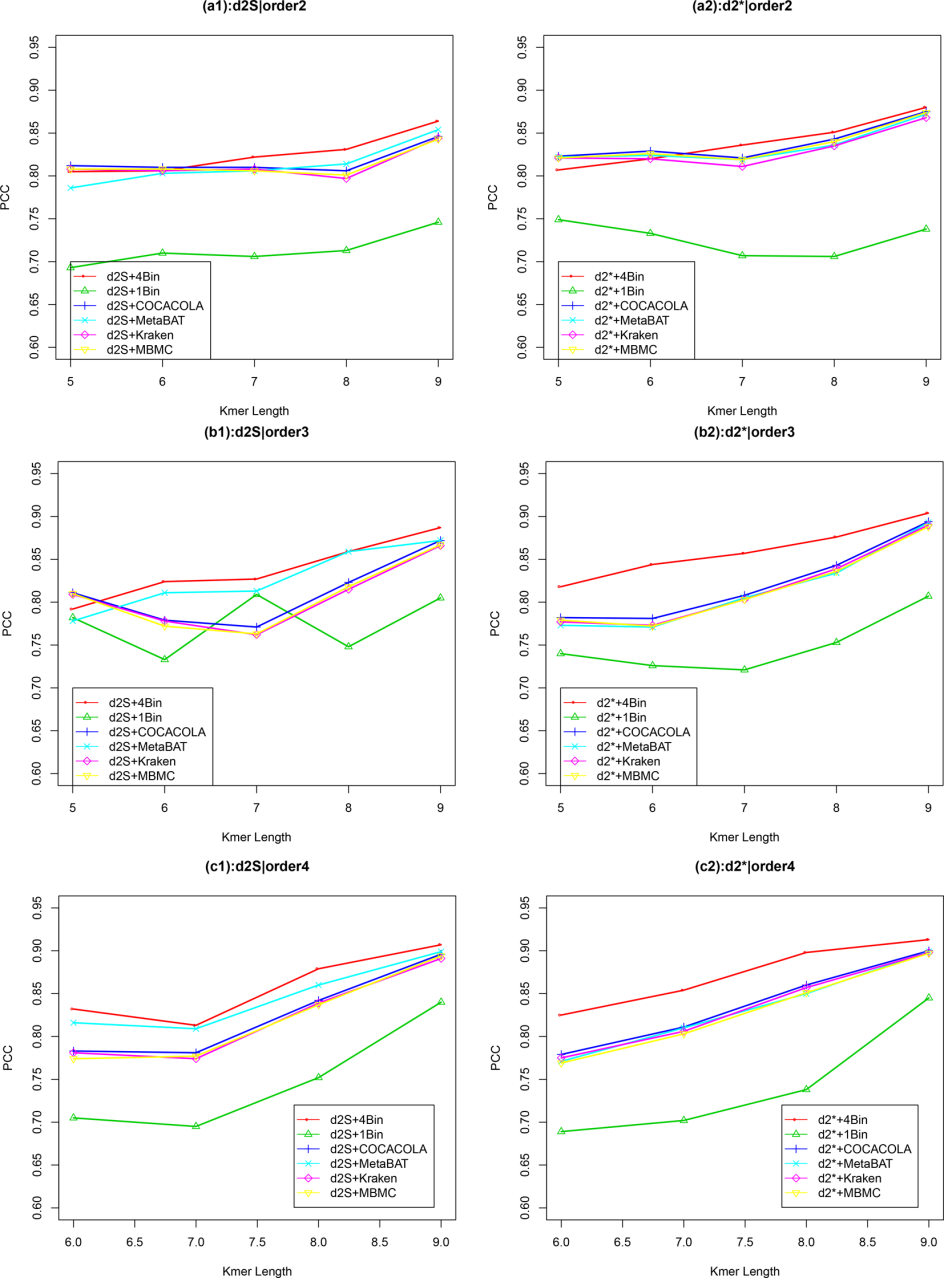
The relative performance (Pearson correlation coefficient) of various reads binning methods in recovering gradient relationships of the metagenomic samples for Simulation 2 at sequencing depth of 500,000 next-generation sequencing paired-end reads. The background sequence Markov orders were two (a1, a2), three (b1, b2) and four (c1, c2). The dissimilarity measures $d_{2}^{S}$ and $d_{2}^{*}$ with binning into 4 bins outperform other binning methods in most situations. The corresponding figures based on Markov order zero and one are presented as **Figure S4** in **Supplementary Material**.

Similar to Simulation 1, we generated two sequencing depths of 100,000 and 500,000 paired-end reads per sample. **Figure 4** showed the average PCC of the different dissimilarity measures at different tuple sizes and Markov orders of background sequences. Similar to the results in Simulation 1, reads binning improved the results compared to no binning for both alignment-free measures, $d_{2}^{S}$ and $d_{2}^{*}$. The PCC values increased with tuple size and Markov order. For a fixed bin number of reads and tuple size, the PCC values increased more than 0.10 from order 0 to order 4, indicating that higher order Markov chains could model the genomic sequences better. The performance of $d_{2}^{*}$ is slightly better than that of $d_{2}^{S}$ for gradient detection. The best result with the largest PCC value was obtained by $d_{2}^{*}$ with reads binning using tuple size *k* = 9 and background Markov order 4. To test if the highest PCC is statistically significantly higher than the second highest PCC, we generated 10 duplicated datasets to simulate possible stochastic effects in real NGS data and obtained the PCC for each duplication. Using paired t-test, the resulting one-sided p-value is less than 0.0005. In **Table 2**, we fixed the tuple size as 9 for $d_{2}^{S}$ and $d_{2}^{*}$, and compared the effect of number of read bins on recovering gradient relationships. Again, results showed that the alignment-free methods without reads binning had the lowest values of PCC, i.e., worst performance, while methods with reads binning into 2 to 5 bins improved performance. For a given order of Markov chain, the PCCs corresponding to binning reads to 3, 4, or 5 bins are similar, indicating that that the number of reads bins does not markedly affect the performance of our methods when the bin number is at least 3. The simulations using a relatively shallow sequencing with 100,000 paired-end reads also gave results similar to those of deeper sequencing with 500,000 paired-end reads (**Figures S4** and **S5**). **Figure S1(c, d)** showed that the PCC values only decreased significantly when the sequencing error was 0.05 suggesting that sequencing errors only slightly impact the performance of the measures. **Figure 4** shows that all these reads binning schemes are better than the original version without any reads binning, but they do not perform as well as the above scheme based on binning from Markov chains.

**Table 2:** The Pearson correlation between the first principal coordinate and the simulated environmental gradient using different numbers of bins for the reads with tuple size *k* = 9 and Markov order from 0 to 4 for Simulation 2 at sequencing depth of 500,000 next-generation sequencing paired-end reads.

		No binning	2 bins	3 bins	4 bins	5 bins
$d_{2}^{S}$	order 0	0.721	0.782	0.791	0.787	0.787
	order 1	0.769	0.855	0.852	0.851	0.849
	order 2	0.746	0.860	0.863	0.864	0.861
	order 3	0.805	0.896	0.893	0.887	0.844
	order 4	0.840	0.899	**0.907**	**0.907**	0.906
$d_{2}^{*}$	order 0	0.617	0.766	0.760	0.757	0.755
	order 1	0.724	0.871	0.870	0.871	0.871
	order 2	0.738	0.887	0.880	0.880	0.880
	order 3	0.807	0.904	0.903	0.904	0.901
	order 4	0.845	0.903	**0.914**	0.913	**0.914**

The two highest Pearson correlations are in boldface.

### Detecting Group Relationships Among Human Gut Samples

We applied the alignment-free methods to analyze human gut metagenomic datasets from different countries. These datasets include 107 fecal microbiome samples from Asia (Kurokawa et al., 2007; Qin et al., 2012), Europe (Qin et al., 2010) and North America (Turnbaugh et al., 2009). Two countries (China and Japan, n = 45 and 13) are from Asia, two countries (Denmark and Spain, n = 21 and 10) are from Europe, and one country (USA, n = 18) is from North America. In the simulation results, we found that the triplet distance and PCC values of the alignment-free dissimilarity measures $d_{2}^{S}$ and $d_{2}^{*}$ could achieve the best performance when the NGS reads were classified to four bins. Consequently, in the real data analysis, we used all the bacterial genomic sequences both before May 2015 and after June 2015 to construct four different Markov Models to bin these NGS reads.

First, we used alignment-free measures, $d_{2}^{S}$ and $d_{2}^{*}$, with tuple size 9 and Markov order 4 to explore the relationship among these human gut metagenomic samples. Similar to the simulation studies, we used UPGMA to cluster the samples based on the dissimilarity matrix, as defined by different dissimilarity measures based on sequence signatures. **Figure S6** showed that these human gut samples could be clustered into four different groups labeled with different colors. The Japanese and American samples could be clearly separated from other groups with no overlaps. Most Chinese and European samples could be grouped separately, but with some overlaps. The samples from Denmark and Spain could not be distinguished from each other. A previous study (Costea et al., 2018) showed that the gut microbial community of both Chinese and European samples was enriched with *Firmicutes, Bacteroides and Prevotella*; however, the American samples all indicated a high-fat diet and were enriched with only *Bacteroides*. Therefore, both Chinese and European samples had similar microbial composition and should first be clustered together and then clustered again with the Japanese samples. The American samples have distinct gut microbial composition and should be separated from other samples.

**Figure 5:**
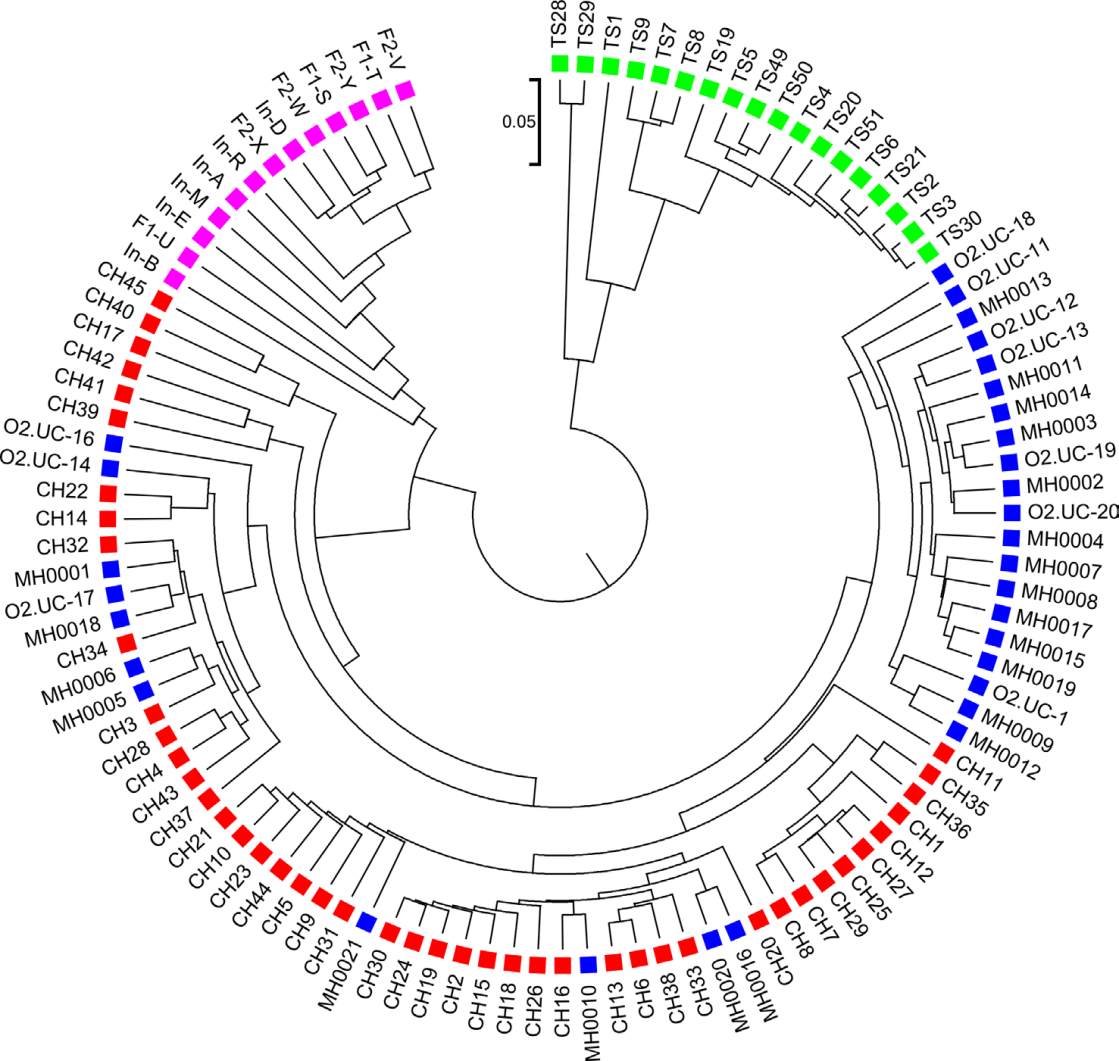
The best clustering tree for the 107 human fecal metagenomic samples based on the newly developed dissimilarity measure $d_{2}^{S}$ with tuple size *k* = 6 and background sequence Markov order = 4. Red squares: Chinese samples; blue squares: European samples; purple squares: Japanese samples; green squares: American samples.

We next calculated the triplet distance based on the four divided groups for $d_{2}^{S}$ and $d_{2}^{*}$. The results of triplet distance scores for the different dissimilarity measures are summarized in **Table 3**. The smallest triplet distance score was achieved with $d_{2}^{S}$ coupled with tuple size *k* = 6 and the fourth order Markov chain model of background sequences. When the order of Markov chains was four, the triplet distances were all lower than 30,000 for tuple size *k* from 6 to 9. In addition, triplet distance decreased with increasing Markov order for any fixed tuple size. The best performance was achieved when tuple size was *k* = 6 or 7 and Markov order = 4, similar to the *k*-tuple in Simulation 1. **Figure 5** showed the cluster tree using UPGMA for $d_{2}^{S}$ with tuple size *k* = 6 and Markov order 4. **Table S5** showed the confusion matrix for $d_{2}^{S}$ with tuple size *k* = 6 and Markov order 4. **Figure S7** showed the PCoA plot of these 107 samples. In this rooted tree, we found that American samples were separated from other samples and that the Japanese samples were separated from the Chinese and European samples. Although some European samples were mixed with the Chinese samples, most European samples clustered together.

**Table 3:** The triplet distance between the reference and the clustering trees for the 107 human fecal metagenomic samples using various reads binning methods with tuple size *k* = 5–9 and background sequence Markov order from 0 to 4.

	*k*	5	6	7	8	9
$d_{2}^{S}$ without reads binning	order 0	39,281	36,237	34,049	32,908	32,192
	order 1	38,129	35,070	33,306	32,455	32,149
	order 2	34,430	32,511	31,631	31,308	31,645
	order 3	32,124	31,154	31,629	31,738	32,162
	order 4	–	29,841	30,576	31,246	32,063
$d_{2}^{S}$ with 4 bins	order 0	36,468	33,781	31,822	30,735	30,335
	order 1	35,568	32,215	30,569	30,114	30,287
	order 2	29,511	29,006	28,556	28,625	29,436
	order 3	31,112	30,130	29,350	29,468	30,256
	order 4	–	**26,890**	**26,962**	28,102	29,587
$d_{2}^{*}$ without reads binning	order 0	49,732	46,565	42,415	37,998	34,036
	order 1	48,002	45,070	41,444	38,009	33,151
	order 2	43,132	40,134	38,055	33,539	32,171
	order 3	39,180	37,056	34,468	32,912	32,183
	order 4	–	34,656	33,829	33,215	33,054
$d_{2}^{*}$ with 4 bins	order 0	46,942	44,312	40,504	36,556	32,285
	order 1	44,447	41,995	38,726	35,658	31,474
	order 2	37,515	35,859	33,896	30,249	30,154
	order 3	38,555	35,964	32,126	30,965	30,689
	order 4	–	31,816	30,064	30,031	30,799

The two lowest triplet distances are in boldface.

### Detecting Group Relationships Among Human Body Sites

**Figure 6:**
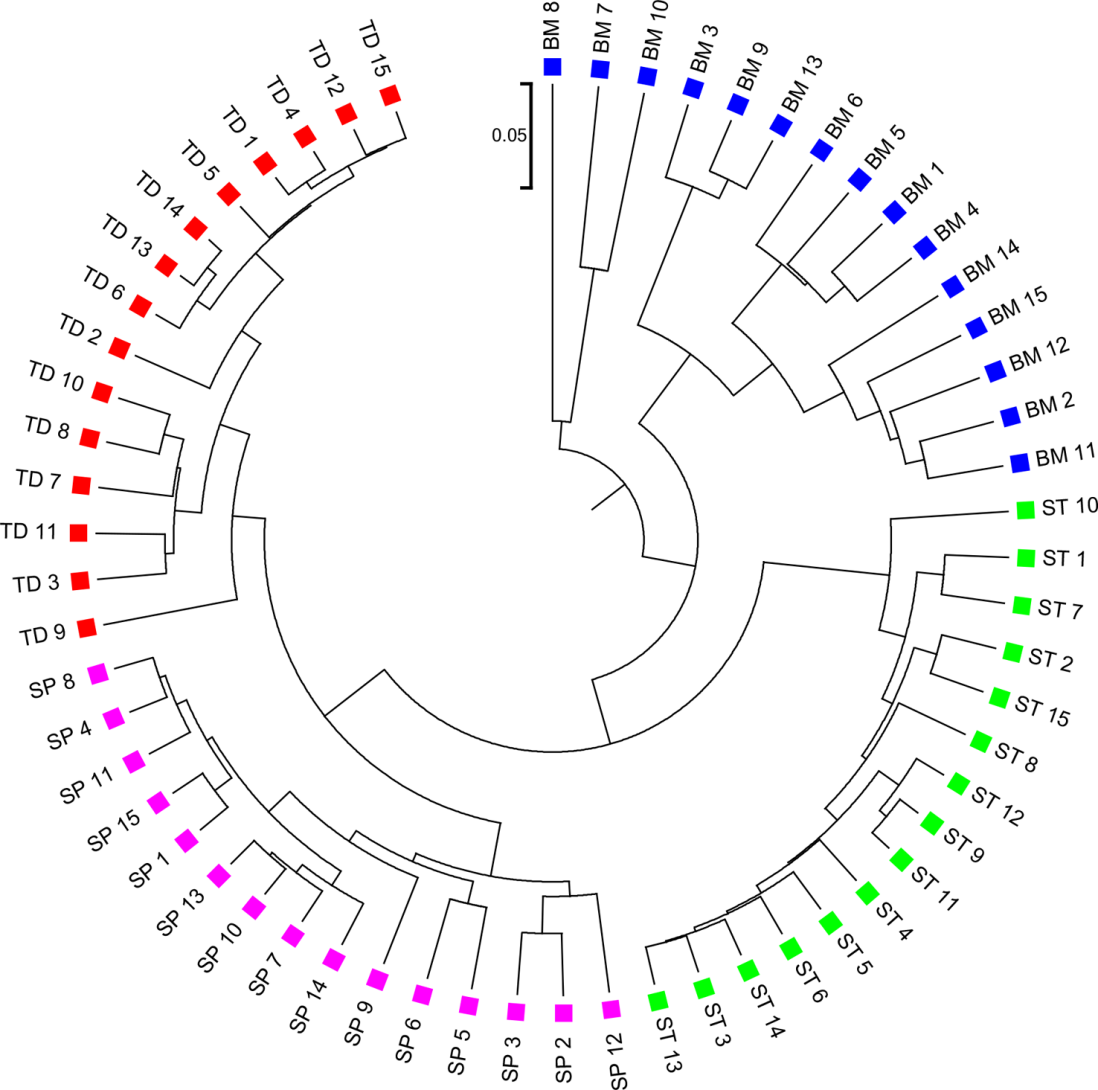
The best clustering tree for the 60 human microbiome samples from four body sites based on newly developed dissimilarity measure $d_{2}^{S}$ with tuple size *k* = 6 and background sequence Markov order = 4. Red squares: Tongue dorsum; Blue squares: Buccal mucosa; Purple squares: Supragingival plaque; Green squares: Stool.

We applied the alignment-free methods to analyze human metagenomic datasets from four body sites: buccal mucosa, supragingival plaque, tongue dorsum, and stool (Lloyd-Price et al., 2017). Each body site had fifteen samples. We calculated the pairwise $d_{2}^{S}$ and $d_{2}^{*}$ dissimilarities for any pair of samples and build a hierarchical clustering tree. We next calculated the triplet distance between the clustering tree with the four divided groups based on body sites. **Table 4** showed that the smallest triplet distance score was achieved with $d_{2}^{S}$ coupled with tuple size *k* = 6 and the fourth order Markov model of background sequences. **Figure 6** showed the cluster tree using UPGMA for $d_{2}^{S}$ with tuple size *k* = 6 and Markov order 4. **Table S6** showed the confusion matrix for $d_{2}^{S}$ with tuple size *k* = 6 and Markov order 4. In this rooted tree, we found that supragingival plaque and tongue dorsum samples were first grouped together and then clustered with the stool samples and buccal mucosa samples, consistent with the results from a previous study (Lloyd-Price et al., 2017).

**Table 4:** The triplet distance between the reference and the clustering trees for the 60 human metagenomic samples across four body sites using various reads binning methods with tuple size *k* = 5–9 and background sequence Markov order from 0 to 4.

	*K*	5	6	7	8	9
$d_{2}^{S}$ without reads binning	order 0	4,536	4,153	3,696	3,306	2,986
	order 1	4,245	3,906	3,887	3,598	3,243
	order 2	3,945	3,657	3,257	3,010	2,798
	order 3	3,116	2,954	2,779	2,638	2,497
	order 4	–	2,215	2,275	2,315	2,382
$d_{2}^{S}$ with 4 bins	order 0	4,342	3,982	4,407	4,073	3,672
	order 1	4,048	3,803	3,544	3,263	3,010
	order 2	3,843	3,541	3,248	3,061	2,868
	order 3	2,960	2,812	2,697	2,573	2,469
	order 4	–	**2,167**	**2,180**	2,206	2,261
$d_{2}^{*}$ without reads binning	order 0	5,281	5,533	6,068	6,419	6,827
	order 1	4,534	5,244	6,069	6,610	6,841
	order 2	4,409	4,744	5,235	5,611	6,254
	order 3	3,800	4,286	5,034	5,861	6,387
	order 4	–	4,057	4,898	5,719	6,269
$d_{2}^{*}$ with 4 bins	order 0	4,640	5,104	5,907	6,436	6,871
	order 1	4,527	5,034	5,837	6,178	6,658
	order 2	4,313	4,978	5,895	6,553	6,879
	order 3	3,496	4,080	4,907	5,836	6,396
	order 4	–	3,823	4,726	5,683	6,315

The two lowest triplet distances are in boldface

### Detecting Group and Gradient Variations in Soil Metagenomic Data

We next applied the alignment-free methods to analyze the metagenomic data of soil microbial communities collected from different geographic locations, spanning a wide range of ecologically distinct biomes, to examine how cold desert soils would compare with hot desert soils, forests, prairie, and tundra (Fierer et al., 2012).

**Figure 7:**
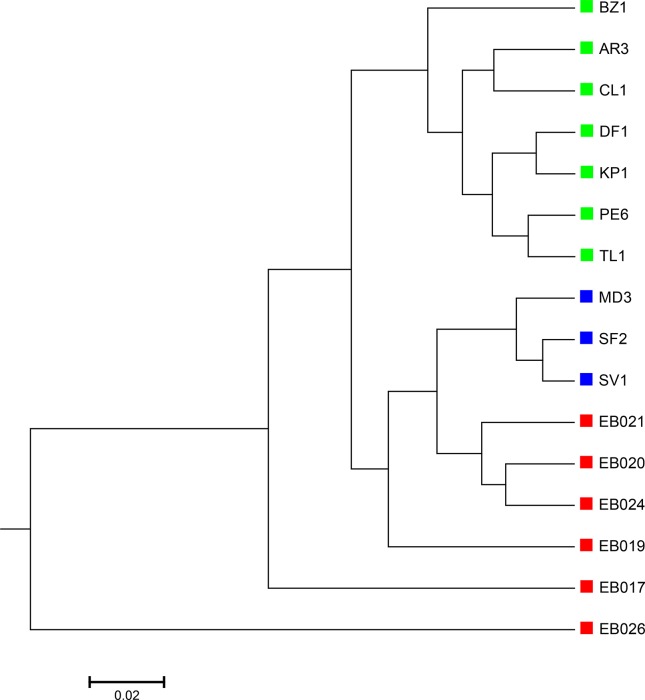
The best clustering tree for the 16 soil metagenomic samples from three ecologically distinct groups based on the newly developed dissimilarity measure $d_{2}^{S}$ coupled with tuple size *k* = 6 and background sequence Markov order = 4. Red squares: polar desert samples; blue squares: hot desert samples; green squares: forest samples.

The 16 soil samples form three ecologically distinct groups: hot deserts (n = 3), cold deserts (n = 6), and worldwide forests (n = 7). We conducted clustering analysis with sequence signatures of these samples and used triplet distance to study how well the grouping information was revealed (**Table 5**). Again, for all tuple size values, it can be seen that the performance of the alignment-free methods improved along with reads binning. Under reads binning, $d_{2}^{*}$ coupled with tuple size *k* = 6 and the fourth order Markov model of background sequences achieved the best performance (**Tables 5** and **S7**, **Figure 7**). We observed that the three major groups identified by the alignment-free methods, $d_{2}^{S}$ and $d_{2}^{*}$, reflected three major ecologically distinct conditions. The main factor that differentiates these soil samples is pH which, in polar and hot deserts, is higher than 7.00, but in worldwide forests lower than 7.00. These three groups of samples had different ranges of pH values. The pH of polar desert ranged from 8.15 to 9.95, while the pH values of hot desert ranged from 7.90 to 8.38. The pH values of worldwide forests ranged from 4.12 to 6.37. In the forest soil samples, the two samples from tropical forest (PE6) and Arctic tundra (TL1) with lowest pH values (4.12 and 4.58) were first clustered together and then clustered again with other forest samples. In order to test whether pH was the main environmental driver of microbial community composition, we tested the correlation between pH values and the first principal coordinate of these samples, and a highly significant negative correlation was found, as shown in **Figure S8** (Pearson correlation = −0.856, p-values = 0.0001). We also examined the correlation among the first to fourth principal coordinate of these samples with other environmental factors, including mean annual precipitation (MAP), mean annual temperature (MAT), organic Carbon content (%C), Nitrogen content (%N), and Carbon : Nitrogen ratio (C:N ratio). The first principal coordinate was also associated with the %C, %N, and C:N ratio (p-values < 0.01). But for the second, third, and fourth principal coordinates, the associations were not significant (**Table S8**).

**Table 5:** The triplet distance between the reference and the clustering trees for the 16 soil metagenomic samples from three ecologically distinct groups using various reads binning methods with tuple size *k* = 5–9 and background sequence Markov order from 0 to 4.

	*k*	5	6	7	8	9
$d_{2}^{S}$ without reads binning	order0	127	121	117	115	115
	order1	110	111	112	113	110
	order2	113	118	116	115	115
	order3	114	113	119	120	123
	order4	–	117	117	118	124
$d_{2}^{S}$ with 4 bins	order0	129	124	124	124	122
	order1	120	121	119	119	118
	order2	114	116	119	121	123
	order3	**108**	111	119	121	123
	order4	–	**108**	117	115	121
$d_{2}^{*}$ without reads binning	order0	115	125	124	120	116
	order1	119	110	111	117	117
	order2	122	120	119	121	141
	order3	124	116	123	136	140
	order4	–	116	130	142	149
$d_{2}^{*}$ with 4 bins	order0	129	126	124	122	116
	order1	122	119	117	119	135
	order2	121	120	120	129	144
	order3	112	112	121	142	143
	order4	–	119	135	145	153

The two lowest triplet scores are in boldface

## Discussion

In this study, we developed new alignment-free measures $d_{2}^{S}$ and $d_{2}^{*}$ for the comparison of metagenomes that model metagenomic reads as from a mixture of multiple Markov chains. We investigated the applications of the new alignment-free measures to compare metagenomic samples. Because of the high complexity of metagenomic data, the previous version of alignment-free measures $d_{2}^{S}$ and $d_{2}^{*}$ in (Jiang et al., 2012) that used only one background Markov model could not capture data heterogeneity. We proposed to first group reads in metagenomic samples into various bins using different Markov models. Then, *k*-tuple frequency vectors were counted and normalized individually in each bin. With the newly developed mixture model for computing the *k*-tuple expectations, we found that the modified $d_{2}^{S}$ and $d_{2}^{*}$ measures with reads binning outperformed the old ones in terms of recovering group and gradient relationships among samples from different environments. We extensively tested the methods on two sets of simulated metagenomic data and two sets of real metagenomic data, including metagenomes of human gut samples and worldwide soil samples. The effects of tuple size *k*, Markov order, and the bin number on the performance of our newly developed alignment-free measures were investigated, and the optimal ranges of those parameters were obtained.

There are several limitations of the current study. First, the performance of the new $d_{2}^{S}$ and $d_{2}^{*}$ measures depends on the number of bins for the reads. In this study, we let the number of bins be 1 to 5 and found that the optimal number of bins for the reads is between 3 and 5 in both simulation and real studies. In practice, we suggest setting the number of bins for the reads as 4. More studies are needed to see if this conclusion is robust for most comparative studies of metagenomic datasets. Second, the tuple size *k* can markedly impact the performance of the new $d_{2}^{S}$ and $d_{2}^{*}$ measures, and the optimal range of *k* can increase with sequencing depth. In general, the tuple size from 6 to 9 can give reasonable results. Third, the optimal range of Markov order is between 3 and 4 in most of our studies. Finally, $d_{2}^{S}$ and $d_{2}^{*}$ have similar performance, but $d_{2}^{S}$ slightly outperforms $d_{2}^{*}$ in most studied scenarios. This result is consistent with the finding that the old version of $d_{2}^{S}$ slightly outperforms the old version of $d_{2}^{*}$ without reads binning.

In this study, we focused on the comparison of metagenomic samples using alignment-free methods with reads binning. However, compared to alignment-based methods for mapping the reads to known genome or pathway databases and then comparing the genome and pathway abundance profiles, alignment-free methods cannot give insights about genomes and pathways responsible for the differences. From this perspective, we can say that alignment-free and alignment-based methods for metagenome comparison complement each other and should be used interactively to understand the dynamics of microbial communities.

## Author Contributions

KS and FS conceived of the project and developed the methods. KS and JR performed the computations. All authors discussed the results and contributed to the final manuscript.

## Funding

The research was supported by the National Natural Science Foundation of China (11701546), U.S. National Institutes of Health (R01GM120624), and National Science Foundation (DMS-1518001).

## Conflict of Interest

The authors declare that the research was conducted in the absence of any commercial or financial relationships that could be construed as a potential conflict of interest.
