# Supplementary material for: Reads Binning Improves Alignment-Free Metagenome Comparison
Source: Front Genet. 2019 Nov 21;10:1156. doi: 10.3389/fgene.2019.01156 (PMC6881972; doi:10.3389/fgene.2019.01156)
Supplement: Supplementary file 1 [file DataSheet_1.docx]

Reads binning improves alignment-free metagenome comparison

Supplementary Material

Kai Song^1,^ ^†,*^, Jie Ren^2,†,§^, Fengzhu Sun^2,*^

^1^School of Mathematics and Statistics, Qingdao University, Qingdao, Shandong, China

^2^Quantitative and Computational Biology Program, University of Southern California, Los Angeles, CA, USA

^†^Equally contributed;

*Corresponding authors ([ksong@qdu.edu.cn](mailto:ksong@qdu.edu.cn), [fsun@usc.edu](mailto:fsun@usc.edu))

§Present address: Google Inc., Mountain View, California, USA


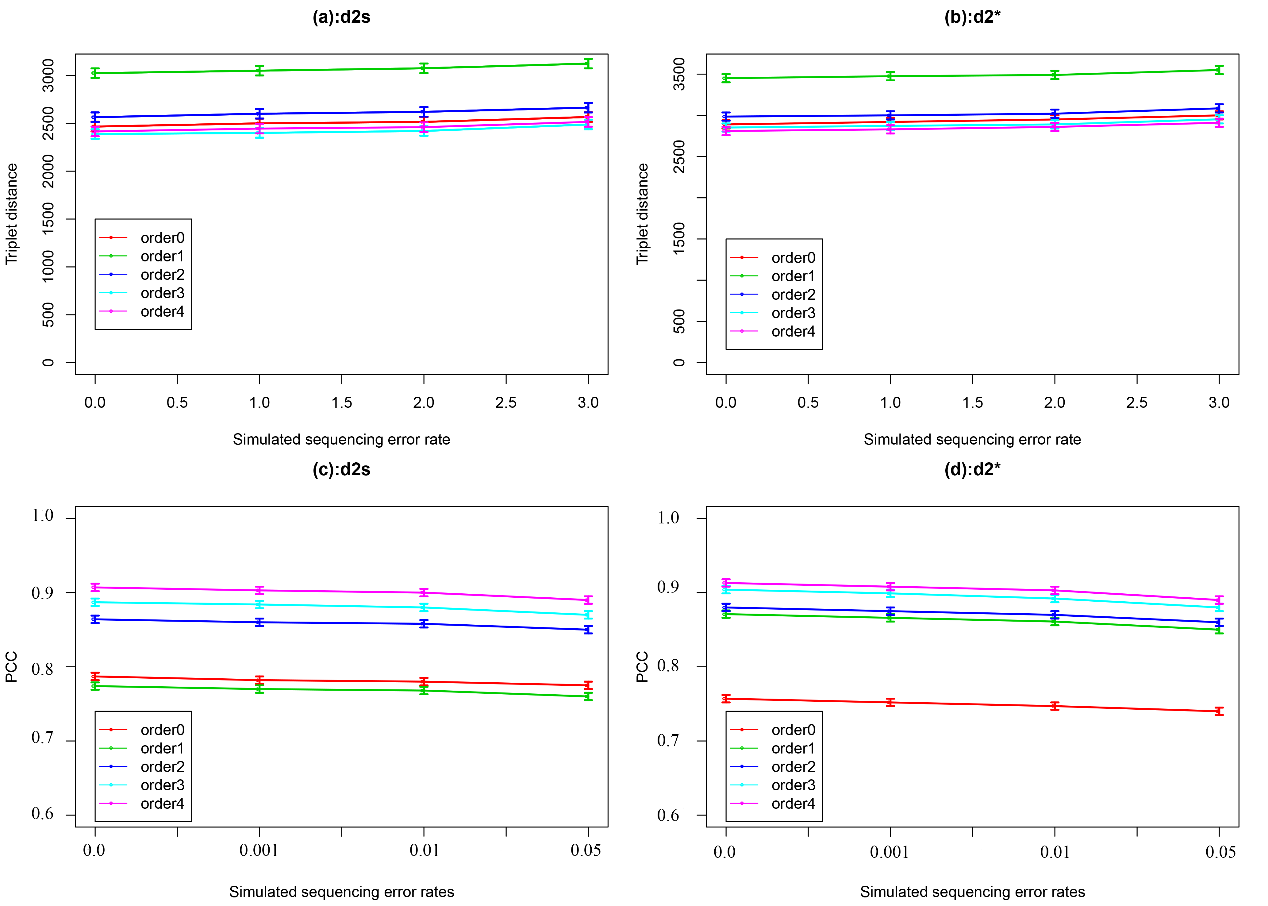


Figure S1. The triplet distances (a,b) and Pearson correlation coefficients (c,d) under different simulated sequencing error rates. For simulation 1 (a,b), the tuple size k = 9 and background sequence Markov order is from 0 to 4 at sequencing depth of 500, 000 NGS paired-end reads. For simulation 2(c,d), the tuple size k = 9 and background sequence Markov order is from 0 to 4 at sequencing depth of 500, 000 NGS pair-end reads.


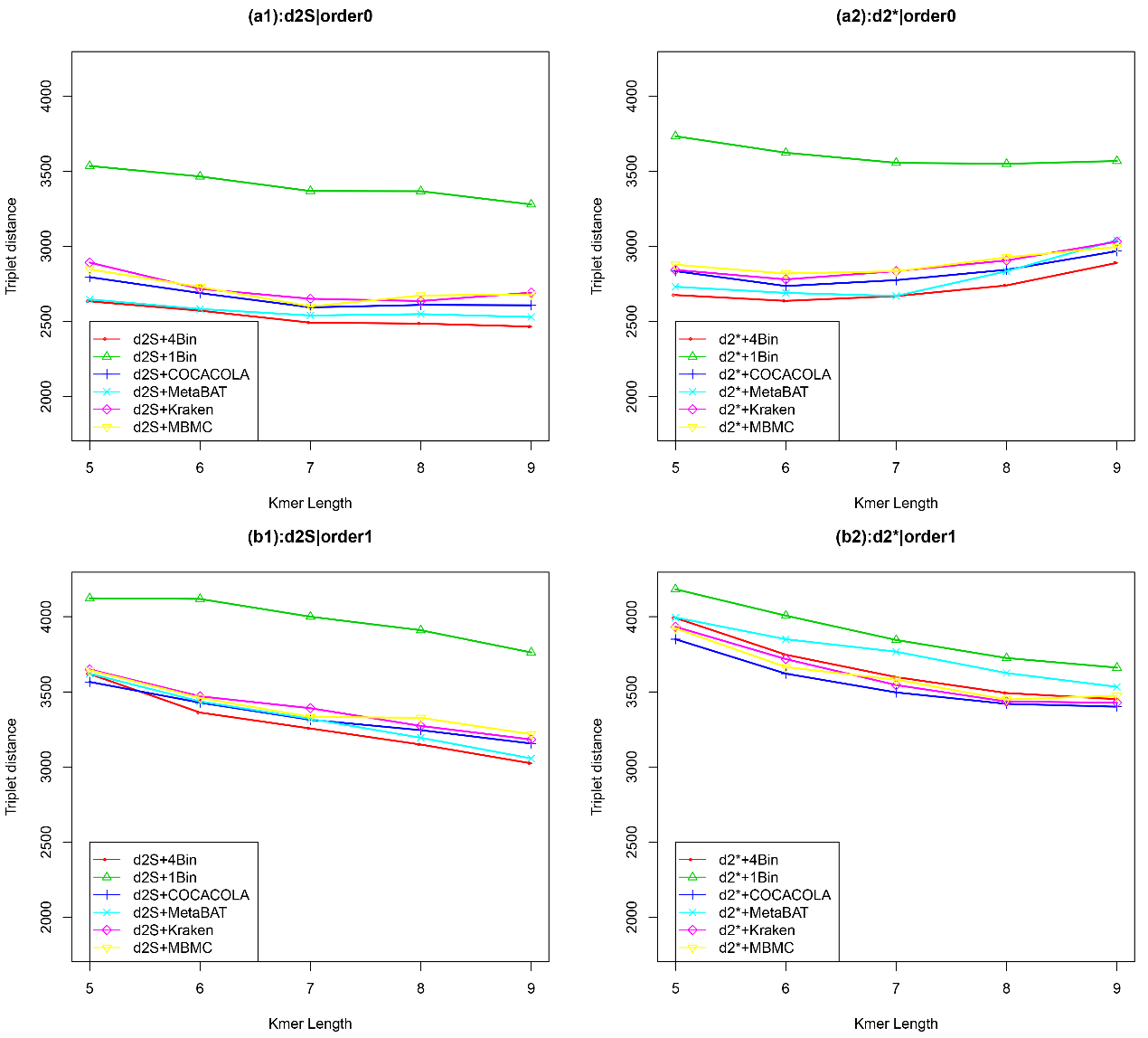


Figure S2. The relative performance (triplet distance) of various reads binning methods in recovering group relationships of the metagenomic samples for Simulation 1 at sequencing depth of 500, 000 NGS paired-end reads. The background sequence Markov orders were zero (a1, a2) and one (b1, b2).


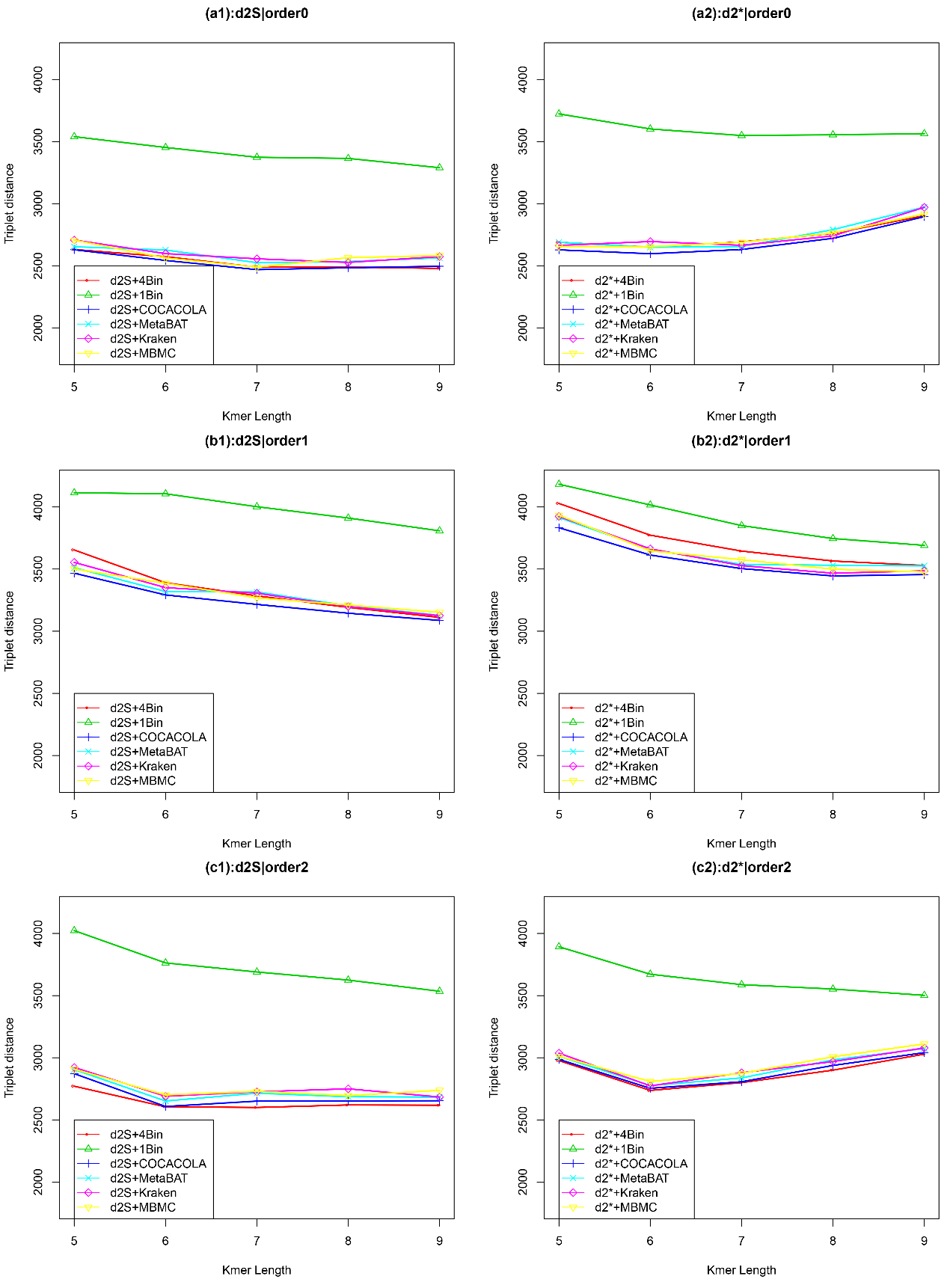


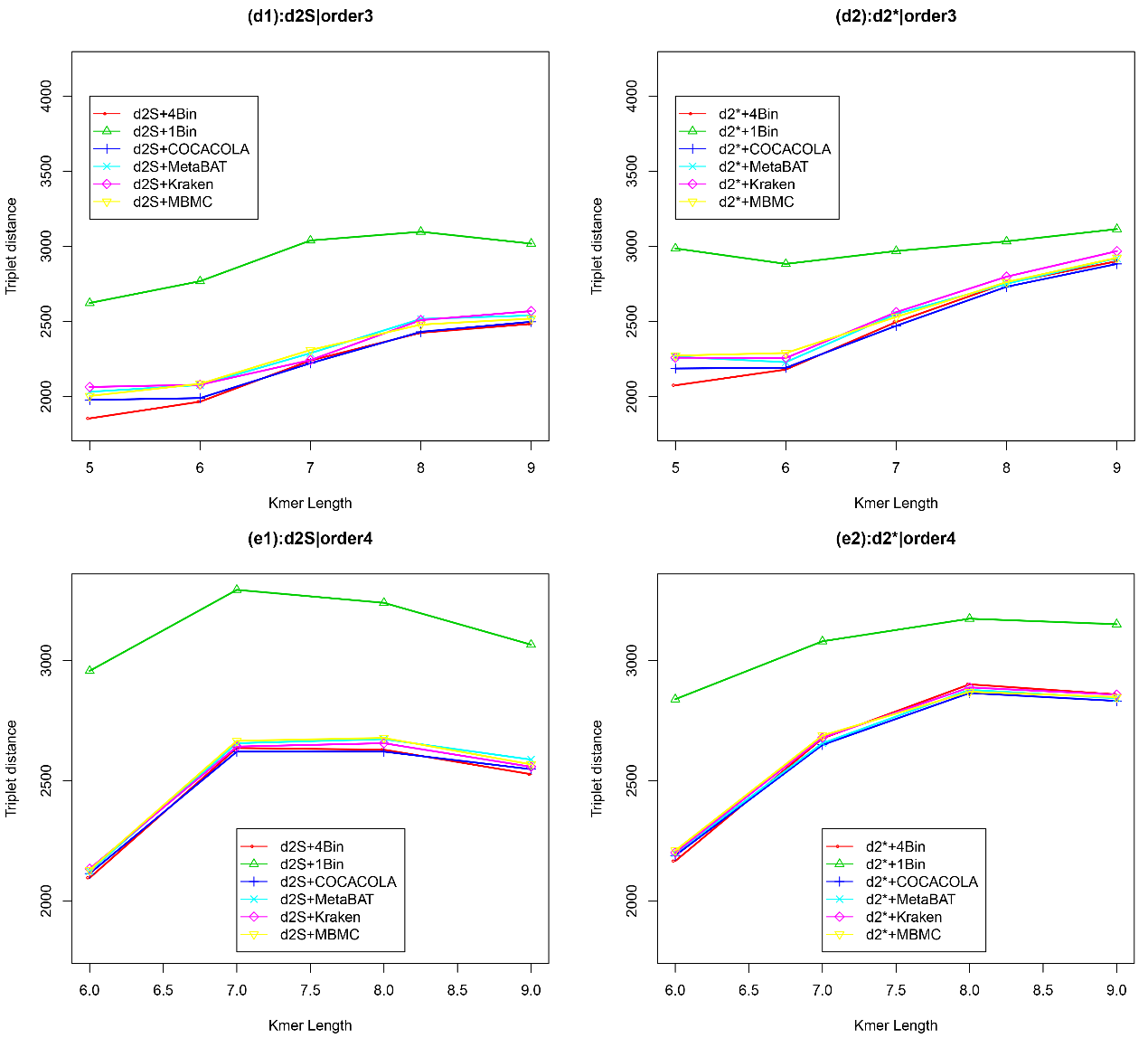


Figure S3. The relative performance (triplet distance) of various reads binning methods in recovering group relationships of the metagenomic samples for Simulation 1 at sequencing depth of 100, 000 NGS paired-end reads. The background sequence Markov orders were zero (a1, a2), one (b1, b2), two (c1, c2), three (d1, d2) and four (e1, e2).


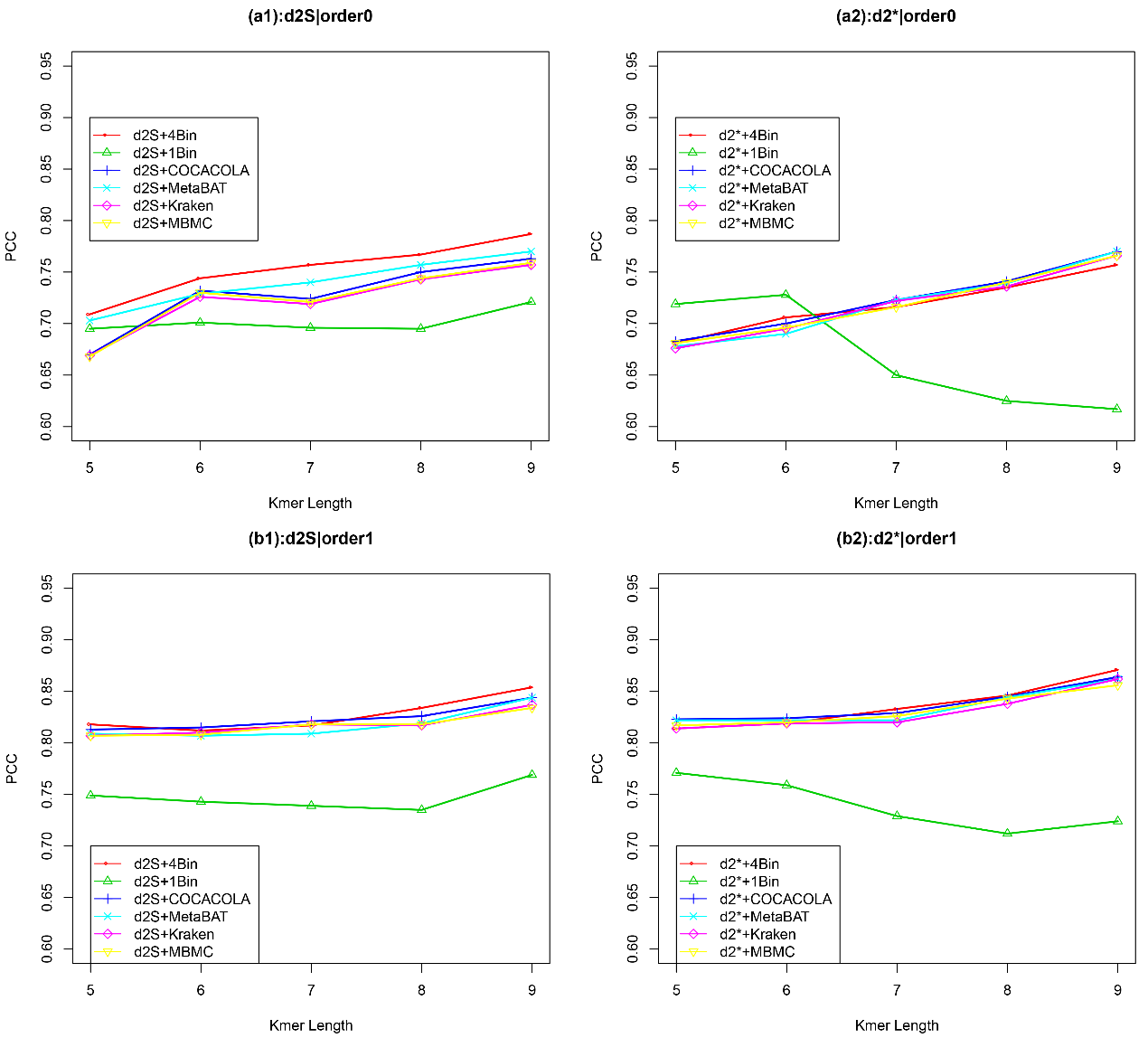


Figure S4. The relative performance (Pearson correlation coefficient) of various reads binning methods in recovering gradient relationships of the metagenomic samples for Simulation 2 at sequencing depth of 500, 000 NGS paired-end reads. The background sequence Markov orders were zero (a1, a2) and one (b1, b2).


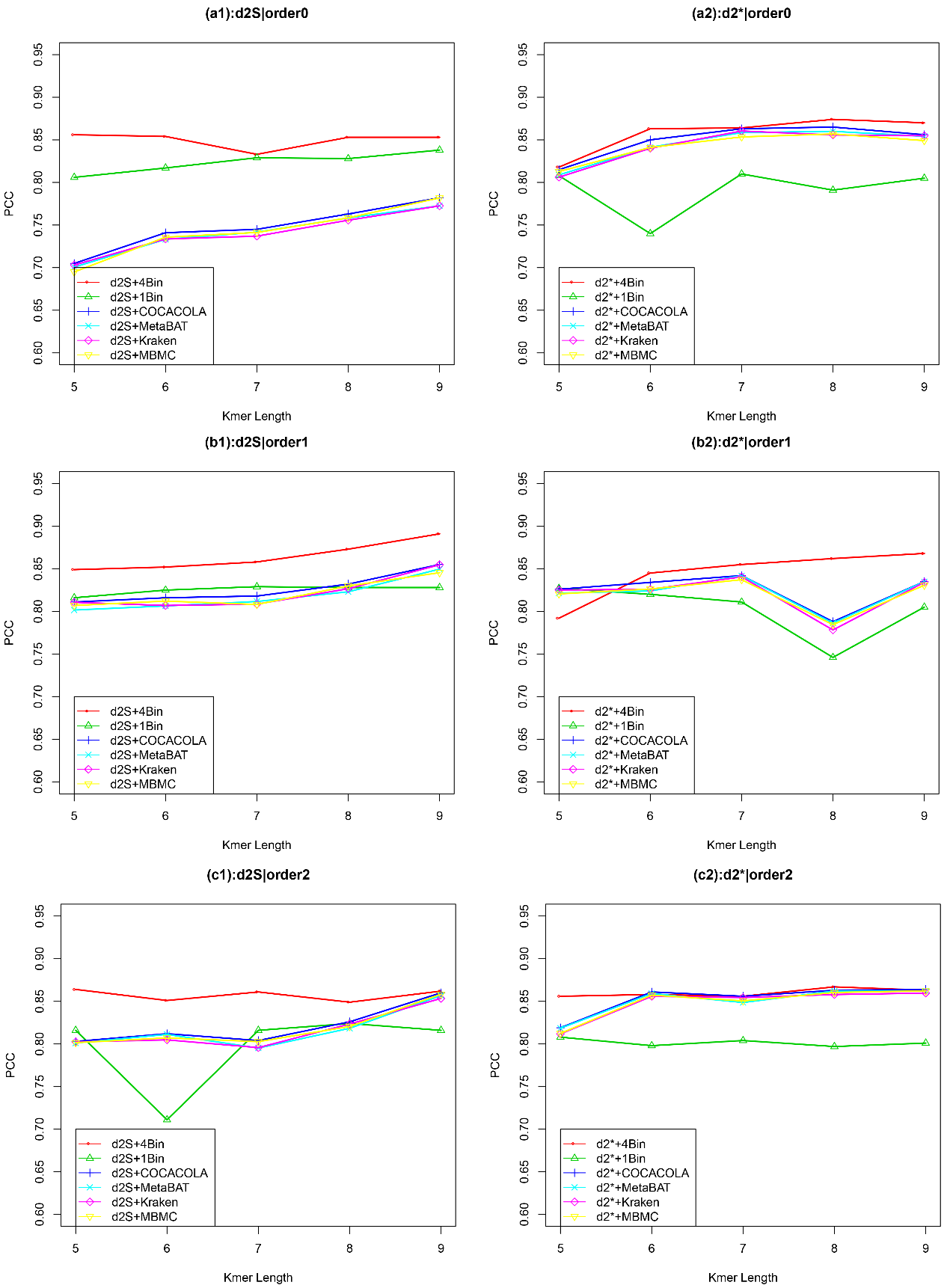


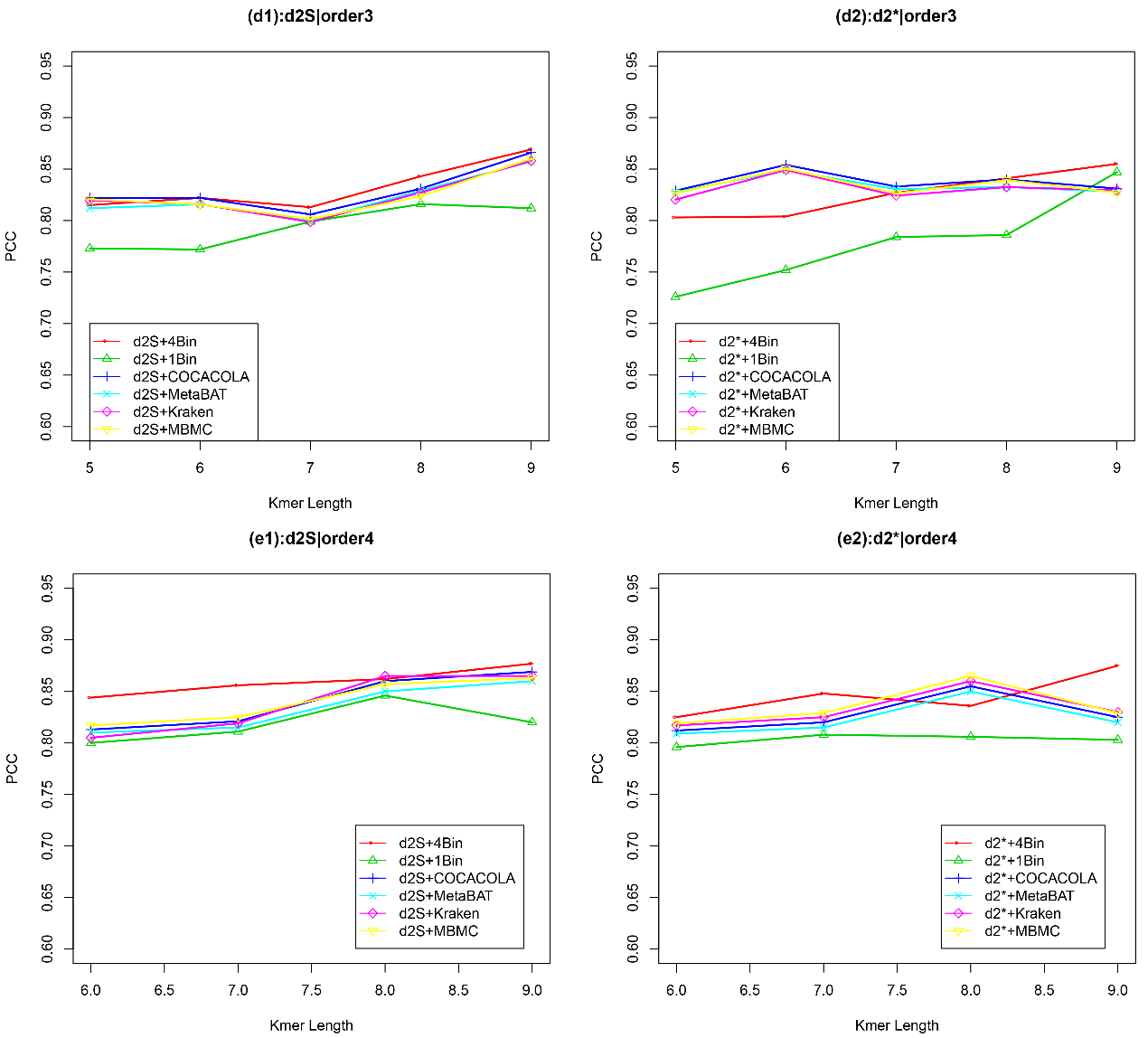


Figure S5. The relative performance (Pearson correlation coefficient) of various reads binning methods in recovering gradient relationships of the metagenomic samples for Simulation 2 at sequencing depth of 100, 000 NGS paired-end reads. The background sequence Markov orders were zero (a1, a2), one (b1, b2), two (c1, c2), three (d1, d2) and four (e1, e2).


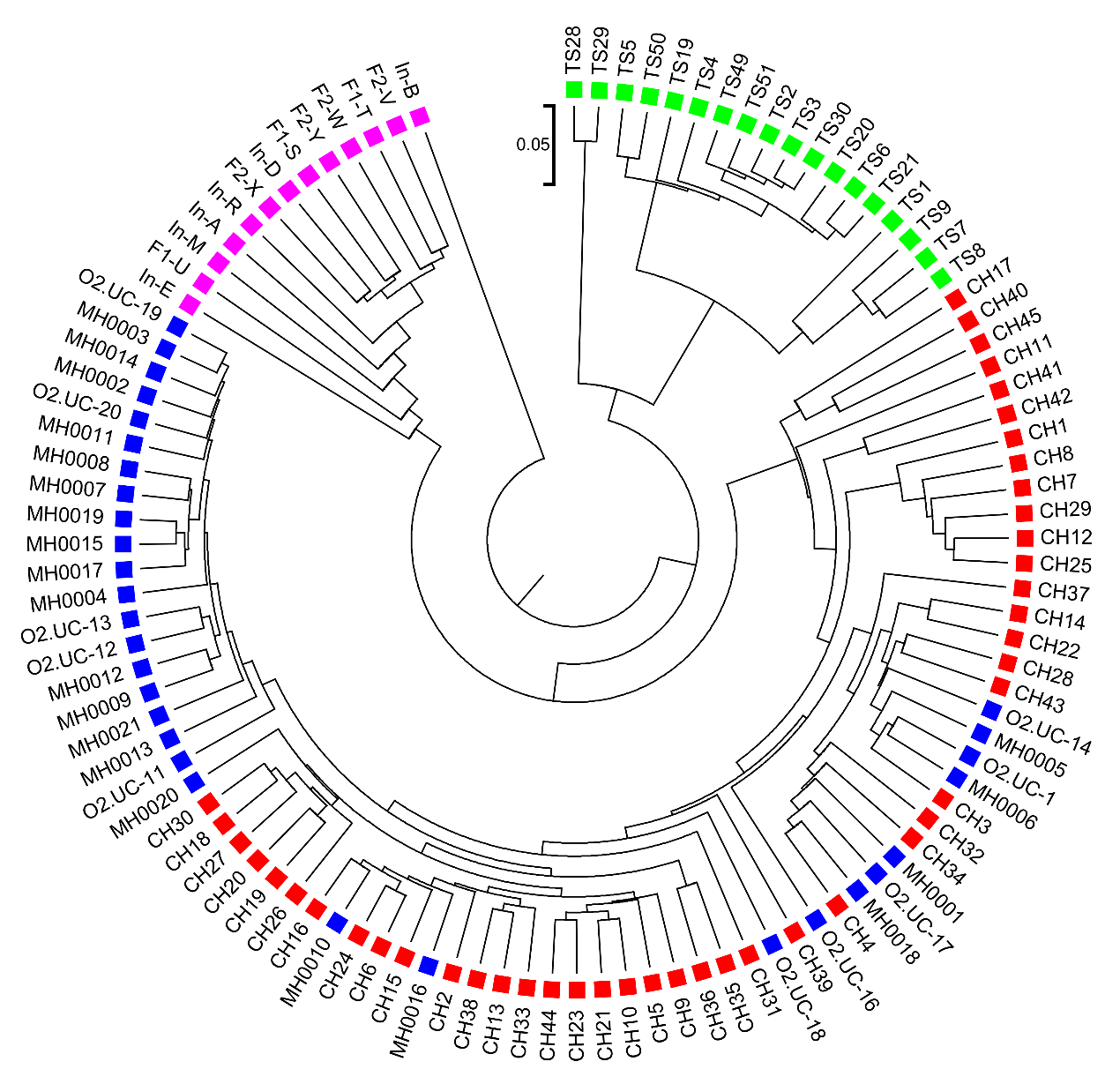


Figure S6. The clustering tree for the 107 human fecal metagenomic samples based on the newly developed dissimilarity measure $d_{2}^{S}$ with tuple size *k* = 9 and background sequence Markov order = 4. Red squares: Chinese samples; blue squares: European samples; purple squares: Japanese samples; green squares: American samples.


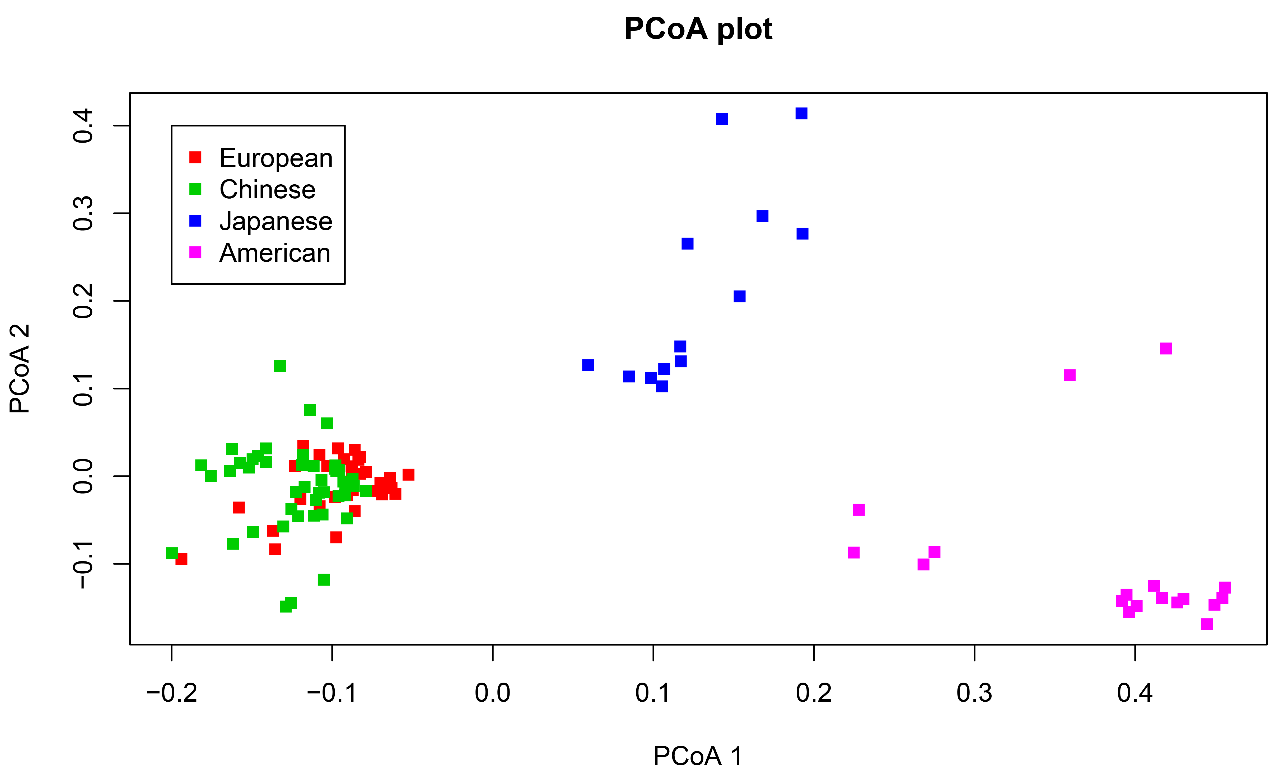


Figure S7: The principal coordinate analysis (PCoA) plot of the 107 human fecal metagenomic samples based on the newly developed dissimilarity measure $d_{2}^{S}$ with tuple size *k* = 6 and background sequence Markov order = 4.


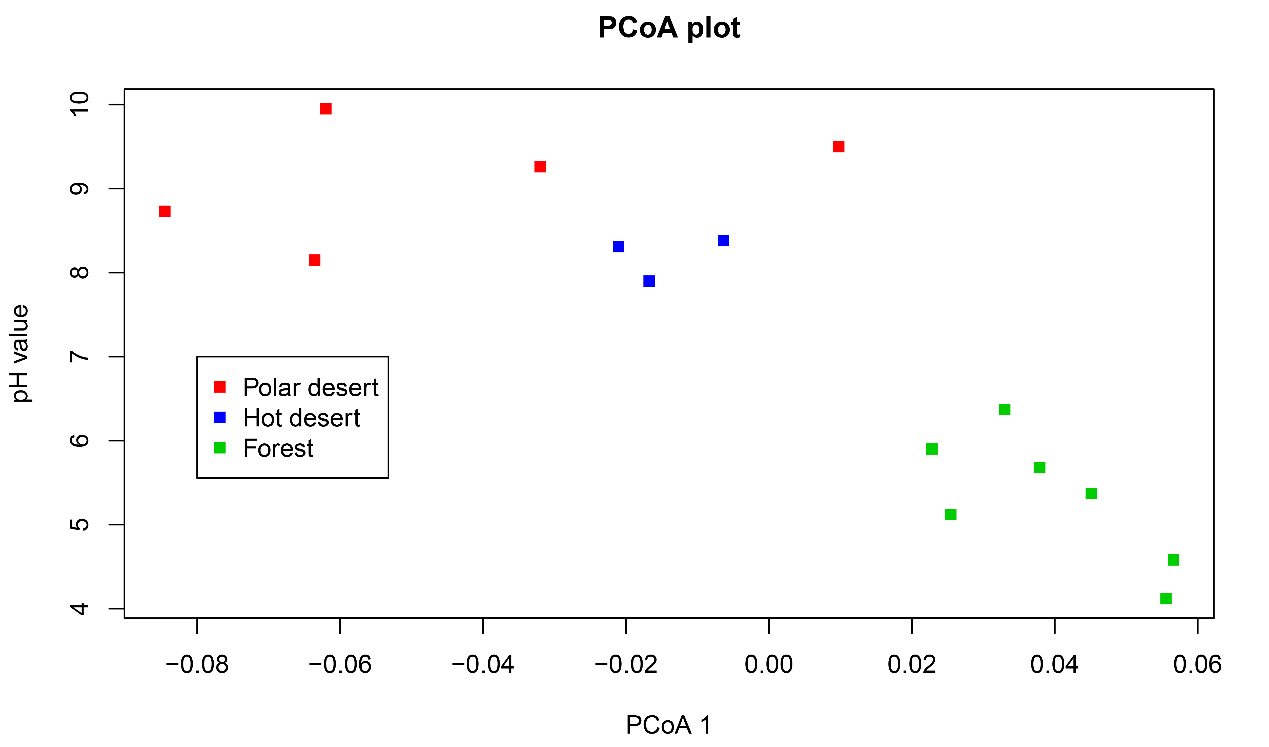


Figure S8: Scatter plot of the soil samples using the first principal coordinate (PCoA 1) and their pH values. One polar sample was far from all other samples and thus could be viewed as an outlier and not used in the figure.

Table S1. List of the 100 bacterial species used in simulation.

| Baterial name | NCBI accessions |
| --- | --- |
| Bacteroides vulgatus ATCC 8482 | NC_009614.1 |
| Bacteroides helcogenes P 36-108 | NC_014933.1 |
| Alistipes finegoldii DSM 17242 | NC_018011.1 |
| Parabacteroides distasonis ATCC 8503 | NC_009615.1 |
| Bacteroides fragilis NCTC 9343 | NC_003228.3 |
| Odoribacter splanchnicus DSM 20712 | NC_015160.1 |
| Bacteroides salanitronis DSM 18170 | NC_015164.1 |
| Roseburia hominis A2-183 | NC_015977.1 |
| Eubacterium rectale ATCC 33656 | NC_012781.1 |
| Akkermansia muciniphila ATCC BAA-835 | NC_010655.1 |
| Clostridium saccharoperbutylacetonicum N1-4(HMT) | NC_020291.1 |
| Chitinophaga pinensis DSM 2588 | NC_013132.1 |
| Niastella koreensis GR20-10 | NC_016609.1 |
| Flavobacterium johnsoniae UW101 | NC_009441.1 |
| Paludibacter propionicigenes WB4 | NC_014734.1 |
| Prevotella ruminicola 23 | NC_014033.1 |
| Clostridium beijerinckii NCIMB 8052 | NC_009617.1 |
| Sphingobacterium sp. 21 | NC_015277.1 |
| Flexibacter litoralis DSM 6794 | NC_018018.1 |
| Cytophaga hutchinsonii ATCC 33406 | NC_008255.1 |
| Clostridium saccharobutylicum DSM 13864 | NC_022571.1 |
| Rivularia sp. PCC 7116 | NC_019678.1 |
| Trichodesmium erythraeum IMS101 | NC_008312.1 |
| Clostridium pasteurianum BC1 | NC_021182.1 |
| Pedobacter saltans DSM 12145 | NC_015177.1 |
| Cyclobacterium marinum DSM 745 | NC_015914.1 |
| Clostridium cellulovorans 743B | NC_014393.1 |
| Solitalea canadensis DSM 3403 | NC_017770.1 |
| Pedobacter heparinus DSM 2366 | NC_013061.1 |
| Prevotella denticola F0289 | NC_015311.1 |
| Haliscomenobacter hydrossis DSM 1100 | NC_015510.1 |
| Clostridium sp. BNL1100 | NC_016791.1 |
| Zunongwangia profunda SM-A87 | NC_014041.1 |
| Sebaldella termitidis ATCC 33386 | NC_013517.1 |
| Runella slithyformis DSM 19594 | NC_015703.1 |
| Nostoc punctiforme PCC 73102 | NC_010628.1 |
| Clostridium lentocellum DSM 5427 | NC_015275.1 |
| Echinicola vietnamensis DSM 17526 | NC_019904.1 |
| Emticicia oligotrophica DSM 17448 | NC_018748.1 |
| Spirosoma linguale DSM 74 | NC_013730.1 |
| Fluviicola taffensis DSM 16823 | NC_015321.1 |
| Marivirga tractuosa DSM 4126 | NC_014759.1 |
| Cellulophaga algicola DSM 14237 | NC_014934.1 |
| Clostridium cellulolyticum H10 | NC_011898.1 |
| Dyadobacter fermentans DSM 18053 | NC_013037.1 |
| Brachyspira hyodysenteriae WA1 | NC_012225.1 |
| Stanieria cyanosphaera PCC 7437 | NC_019748.1 |
| Calothrix sp. PCC 6303 | NC_019751.1 |
| Clostridium botulinum E3 str. Alaska E43 | NC_010723.1 |
| Desulfobacula toluolica Tol2 complete genome | NC_018645.1 |
| Psychroflexus torquis ATCC 700755 | NC_018721.1 |
| Anabaena cylindrica PCC 7122 | NC_019771.1 |
| Cylindrospermum stagnale PCC 7417 | NC_019757.1 |
| Gramella forsetii KT0803 complete circular genome | NC_008571.1 |
| Brachyspira intermedia PWS/A | NC_017243.1 |
| Polaribacter sp. MED152 | NC_020830.1 |
| Oscillatoria acuminata PCC 6304 | NC_019693.1 |
| Brachyspira murdochii DSM 12563 | NC_014150.1 |
| Cyanothece sp. PCC 7424 | NC_011729.1 |
| Microcoleus sp. PCC 7113 | NC_019738.1 |
| Calothrix sp. PCC 7507 | NC_019682.1 |
| Oscillatoria nigro-viridis PCC 7112 | NC_019729.1 |
| Cyanothece sp. PCC 7822 | NC_014501.1 |
| Alkaliphilus metalliredigens QYMF | NC_009633.1 |
| Flavobacterium columnare ATCC 49512 | NC_016510.2 |
| Paenibacillus sp. JDR-2 | NC_012914.1 |
| Flavobacterium indicum GPTSA100-9 complete genome | NC_017025.1 |
| Bacillus sp. 1NLA3E | NC_021171.1 |
| Bacillus infantis NRRL B-14911 | NC_022524.1 |
| Ignavibacterium album JCM 16511 | NC_017464.1 |
| Belliella baltica DSM 15883 | NC_018010.1 |
| Nostoc sp. PCC 7107 | NC_019676.1 |
| Bacillus cellulosilyticus DSM 2522 | NC_014829.1 |
| Cellulophaga lytica DSM 7489 | NC_015167.1 |
| Colwellia psychrerythraea 34H | NC_003910.7 |
| Desulfosporosinus orientis DSM 765 | NC_016584.1 |
| Leadbetterella byssophila DSM 17132 | NC_014655.1 |
| Photorhabdus luminescens subsp. laumondii TTO1 complete genome | NC_005126.1 |
| Arcobacter nitrofigilis DSM 7299 | NC_014166.1 |
| Nonlabens dokdonensis DSW-6 | NC_020156.1 |
| Acetobacterium woodii DSM 1030 | NC_016894.1 |
| Flavobacterium branchiophilum FL-15 | NC_016001.1 |
| Clostridium ljungdahlii DSM 13528 | NC_014328.1 |
| Cyanobacterium aponinum PCC 10605 | NC_019776.1 |
| Eubacterium limosum KIST612 | NC_014624.2 |
| Clostridium botulinum BKT015925 | NC_015425.1 |
| Desulfosporosinus acidiphilus SJ4 | NC_018068.1 |
| Nostoc sp. PCC 7524 | NC_019684.1 |
| Paenibacillus sp. Y412MC10 | NC_013406.1 |
| Lacinutrix sp. 5H-3-7-4 | NC_015638.1 |
| Clostridium novyi NT | NC_008593.1 |
| Halanaerobium praevalens DSM 2228 | NC_017455.1 |
| Bacillus weihenstephanensis KBAB4 | NC_010184.1 |
| Treponema succinifaciens DSM 2489 | NC_015385.1 |
| Lysinibacillus sphaericus C3-41 | NC_010382.1 |
| Desulfovibrio salexigens DSM 2638 | NC_012881.1 |
| Capnocytophaga ochracea DSM 7271 | NC_013162.1 |
| Aequorivita sublithincola DSM 14238 | NC_018013.1 |
| Bacillus cytotoxicus NVH 391-98 | NC_009674.1 |
| Muricauda ruestringensis DSM 13258 | NC_015945.1 |

Table S2. The Accession Number for Human gut datasets.

| Chinese Sample |  | SRA Accession Number | | Europen Sample | | ERR Accession Number |
| --- | --- | --- | --- | --- | --- | --- |
| CH1 |  | SRR341581 | | MH0001 | | ERR011087 |
| CH2 |  | SRR341582 | | MH0002 | | ERR011089 |
| CH3 |  | SRR341583 | | MH0003 | | ERR011092 |
| CH4 |  | SRR341584 | | MH0004 | | ERR011095 |
| CH5 |  | SRR341585 | | MH0005 | | ERR011097 |
| CH6 |  | SRR341586 | | MH0006 | | ERR011099 |
| CH7 |  | SRR341587 | | MH0007 | | ERR011105 |
| CH8 |  | SRR341588 | | MH0008 | | ERR011107 |
| CH9 |  | SRR341589 | | MH0009 | | ERR011109 |
| CH10 |  | SRR341590 | | MH0010 | | ERR011113 |
| CH11 |  | SRR341591 | | MH0011 | | ERR011115 |
| CH12 |  | SRR341592 | | MH0012 | | ERR011118 |
| CH13 |  | SRR341593 | | MH0013 | | ERR011124 |
| CH14 |  | SRR341594 | | MH0014 | | ERR011126 |
| CH15 |  | SRR341595 | | MH0015 | | ERR011129 |
| CH16 |  | SRR341596 | | MH0016 | | ERR011131 |
| CH17 |  | SRR341597 | | MH0017 | | ERR011134 |
| CH18 |  | SRR341598 | | MH0018 | | ERR011136 |
| CH19 |  | SRR341616 | | MH0019 | | ERR011138 |
| CH20 |  | SRR341617 | | MH0020 | | ERR011140 |
| CH21 |  | SRR341618 | | MH0021 | | ERR011142 |
| CH22 |  | SRR341619 | | O2.UC-1 | | ERR011273 |
| CH23 |  | SRR341620 | | O2.UC-11 | | ERR011275 |
| CH24 |  | SRR341621 | | O2.UC-12 | | ERR011277 |
| CH25 |  | SRR341622 | | O2.UC-13 | | ERR011279 |
| CH26 |  | SRR341623 | | O2.UC-14 | | ERR011281 |
| CH27 |  | SRR341624 | | O2.UC-16 | | ERR011283 |
| CH28 |  | SRR341625 | | O2.UC-17 | | ERR011285 |
| CH29 |  | SRR341626 | | O2.UC-18 | | ERR011287 |
| CH30 |  | SRR341627 | | O2.UC-19 | | ERR011289 |
| CH31 |  | SRR341628 | | O2.UC-20 | | ERR011291 |
| CH32 |  | SRR341629 | |  | |  |
| CH33 |  | SRR341630 | |  | |  |
| CH34 |  | SRR341631 | |  | |  |
| CH35 |  | SRR341632 | |  | |  |
| CH36 |  | SRR341633 | |  | |  |
| CH37 |  | SRR341634 | |  | |  |
| CH38 |  | SRR341635 | |  | |  |
| CH39 |  | SRR341654 | |  | |  |
| CH40 |  | SRR341655 | |  | |  |
| CH41 |  | SRR341657 | |  | |  |
| CH42 |  | SRR341660 | |  | |  |
| CH43 |  | SRR341695 | |  | |  |
| CH44 |  | SRR341700 | |  | |  |
| CH45 |  | SRR341703 | |  | |  |
| Japanese Sample | | | Accession numbers | |  |  |
| F1-S | | | BAAU01000001-BAAU01028900 | |  |  |
| F1-T | | | BAAV01000001-BAAV01036326 | |  |  |
| F1-U | | | BAAW01000001-BAAW01016539 | |  |  |
| F2-V | | | BAAX01000001-BAAX01036455 | |  |  |
| F2-W | | | BAAY01000001-BAAY01030198 | |  |  |
| F2-X | | | BAAZ01000001-BAAZ01031237 | |  |  |
| F2-Y | | | BABA01000001-BABA01035177 | |  |  |
| In-A | | | BABB01000001-BABB01020226 | |  |  |
| In-B | | | BABC01000001-BABC01009958 | |  |  |
| In-D | | | BABD01000001-BABD01037296 | |  |  |
| In-E | | | BABE01000001-BABE1020532 | |  |  |
| In-M | | | BABF01000001-BABF01016164 | |  |  |
| In-R | | | BABG01000001-BABG01034797 | |  |  |
| American Sample | | | SRA Accession Number | |  |  |
| TS1 | | | SRX001342 | |  |  |
| TS2 | | | SRX001343 | |  |  |
| TS3 | | | SRX001344 | |  |  |
| TS4 | | | SRX001345 | |  |  |
| TS5 | | | SRX001346 | |  |  |
| TS6 | | | SRX001347 | |  |  |
| TS7 | | | SRX001348 | |  |  |
| TS8 | | | SRX001349 | |  |  |
| TS9 | | | SRX001350 | |  |  |
| TS19 | | | SRX001351 | |  |  |
| TS20 | | | SRX001352 | |  |  |
| TS21 | | | SRX001353 | |  |  |
| TS28 | | | SRX001354 | |  |  |
| TS29 | | | SRX001355 | |  |  |
| TS30 | | | SRX001356 | |  |  |
| TS49 | | | SRX001357 | |  |  |
| TS50 | | | SRX001358 | |  |  |
| TS51 | | | SRX001359 | |  |  |

Table S3. The Accession Number for Human Microbiome Datasets.

| Buccal mucosa | Tongue dorsum | Posterior fornix |
| --- | --- | --- |
| SRR2241599 | SRR2241598 | SRR2241592 |
| SRR2241512 | SRR2241594 | SRR2241511 |
| SRR2241310 | SRR2241506 | SRR1952583 |
| SRR2241214 | SRR2241216 | SRR2241024 |
| SRR2241209 | SRR2241208 | SRR1952519 |
| SRR2241115 | SRR2240958 | SRR2175824 |
| SRR1755757 | SRR2240885 | SRR2175803 |
| SRR1755752 | SRR2240749 | SRR2175799 |
| SRR2240919 | SRR2240575 | SRR2175761 |
| SRR1755747 | SRR2175793 | SRR1952599 |
| Supragingival plaque | Anterior nares | Stool |
| SRR2241513 | SRR2241112 | SRR2241306 |
| SRR2241508 | SRR2241110 | SRR2241118 |
| SRR2241312 | SRR2241025 | SRR2241109 |
| SRR2241218 | SRR1952439 | SRR2240921 |
| SRR2241116 | SRR2240256 | SRR2240917 |
| SRR2241108 | SRR2175760 | SRR2240837 |
| SRR2241021 | SRR1952594 | SRR2240728 |
| SRR2240920 | SRR1952576 | SRR2240291 |
| SRR2175784 | SRR1952563 | SRR2240287 |
| SRR2175748 | SRR1952555 | SRR2240257 |

Table S4. The MG-RAST ID for Soil metagenomic datasets.

| Sample ID | MG-RAST ID |
| --- | --- |
| EB017 | 4477900 |
| EB019 | 4477901 |
| EB020 | 4477902 |
| EB021 | 4477903 |
| EB024 | 4477904 |
| EB026 | 4477803 |
| MD3 | 4477805 |
| SF2 | 4477872 |
| SV1 | 4477873 |
| AR3 | 4477875 |
| BZ1 | 4477876 |
| CL1 | 4477877 |
| DF1 | 4477899 |
| KP1 | 4477804 |
| PE6 | 4477807 |
| TL1 | 4477874 |

Table S5. The confusion matrix for the 107 human fecal metagenomic samples based on the newly developed dissimilarity measure $d_{2}^{S}$ with tuple size *k* = 6 and background sequence Markov order = 4.

|  | Japanese | American | Chinese | European |
| --- | --- | --- | --- | --- |
| Group1 | 13 | 0 | 0 | 0 |
| Group2 | 0 | 18 | 0 | 0 |
| Group3 | 0 | 0 | 45 | 11 |
| Group4 | 0 | 0 | 0 | 20 |

Table S6. The confusion matrix for the 60 human microbiome samples from four body sites based on newly developed dissimilarity measure $d_{2}^{S}$ with tuple size *k* = 6 and background sequence Markov order = 4.

|  | Tongue dorsum | Buccal mucosa | Supragingival plaque | Stool |
| --- | --- | --- | --- | --- |
| Group1 | 15 | 0 | 0 | 0 |
| Group2 | 0 | 15 | 0 | 0 |
| Group3 | 0 | 0 | 15 | 0 |
| Group4 | 0 | 0 | 0 | 15 |

Table S7. The confusion matrix for the 16 soil metagenomic samples from three ecologically distinct groups based on the newly developed dissimilarity measure $d_{2}^{S}$ coupled with tuple size *k* = 6 and background sequence Markov order = 4.

|  | Polar desert | Hot desert | Forest |
| --- | --- | --- | --- |
| Group1 | 6 | 0 | 0 |
| Group2 | 0 | 3 | 0 |
| Group3 | 0 | 0 | 7 |

Table S8. The Pearson correlation coefficients between the principle coordinates and different environmental factors. The significantly associations were bolded.

|  | pH | MAT | MAP | %C | %N | C:N ratio |
| --- | --- | --- | --- | --- | --- | --- |
| PCoA1 | **-0.856** | 0.581 | 0.596 | **0.793** | **0.733** | **0.653** |
| PCoA2 | 0.193 | -0.400 | -0.218 | -0.137 | -0.190 | 0.102 |
| PCoA3 | 0.336 | 0.143 | -0.224 | -0.146 | -0.141 | -0.181 |
| PCoA4 | 0.318 | -0.441 | -0.251 | -0.351 | -0.380 | -0.390 |
